# *ALD5, PAD1, ATF1 and ATF2* facilitate the catabolism of coniferyl aldehyde, ferulic acid and p-coumaric acid in *Saccharomyces cerevisiae*

**DOI:** 10.1038/srep42635

**Published:** 2017-02-16

**Authors:** Peter Temitope Adeboye, Maurizio Bettiga, Lisbeth Olsson

**Affiliations:** 1 Department of Biology and Biological Engineering, Division of Industrial Biotechnology, Chalmers University of Technology, SE-412 96 Gothenburg, Sweden

## Abstract

The ability of *Saccharomyces cerevisiae* to catabolize phenolic compounds remains to be fully elucidated. Conversion of coniferyl aldehyde, ferulic acid and p-coumaric acid by *S. cerevisiae* under aerobic conditions was previously reported. A conversion pathway was also proposed. In the present study, possible enzymes involved in the reported conversion were investigated. Aldehyde dehydrogenase Ald5, phenylacrylic acid decarboxylase Pad1, and alcohol acetyltransferases Atf1 and Atf2, were hypothesised to be involved. Corresponding genes for the four enzymes were overexpressed in a *S. cerevisiae* strain named *APT_1*. The ability of *APT_1* to tolerate and convert the three phenolic compounds was tested. *APT_1* was also compared to strains *B_CALD* heterologously expressing coniferyl aldehyde dehydrogenase from *Pseudomonas*, and an *ald5Δ* strain, all previously reported. *APT_1* exhibited the fastest conversion of coniferyl aldehyde, ferulic acid and p-coumaric acid. Using the intermediates and conversion products of each compound, the catabolic route of coniferyl aldehyde, ferulic acid and p-coumaric acid in *S. cerevisiae* was studied in greater detail.

Lignin complexes and aromatic compounds are abundant organic compounds in nature, second only to cellulose^1^,^2^.

The production of fuels and chemicals from plant biomass require deconstruction and hydrolysis of the biomass before conversion to products of interest^3^. Hydrolysis of plant biomass facilitates the breakdown of cellulose, hemicellulose and lignin in wood, yielding fermentable sugars as well as several biologically active compounds such as phenolic compounds from lignin^4^. Phenolic compounds inhibit the efficient bioconversion of lignocellulose biomass by fermentative organisms. Although the ability of *S. cerevisiae* to degrade some phenolic compounds has been reported^5^, increased phenolics degrading capability in *S. cerevisiae* would enhance its use in the bioconversion of lignocellulosic substrates into fuels and chemicals. Efforts have been made to develop strains of *S. cerevisiae* that possess increased tolerance to some phenolic compounds by identifying and heterologously expressing genes and enzymes of interest, such as laccases^6^,^7^. It is necessary to first identify the genes and enzymes involved and understand their roles in the catabolic process before designing a suitable metabolic engineering strategy. In this line, phenyl acrylic acid decarboxylase (*PAD1*) was found to decarboxylate cinnamic acids in *Saccharomyces cerevisiae*^8^,^9^. In *Pseudomonas sp.* strain HR199 and *Corynebacterium*, the conversion of eugenol to protocatechuic acid, was shown to involve multiple enzymes mechanisms such as the use of oxidoreductases^10^,^11^,^12^.

Of the numerous phenolic compounds in hydrolysates, coniferyl aldehyde (CA) is outstandingly inhibitory to *S. cerevisiae.* In earlier studies, we demonstrated the ability of *S. cerevisiae* to convert CA to several other phenolic metabolites at low concentrations^5^. A *S. cerevisiae* strain with increased resistance to, and a more efficient conversion of CA and other phenolic compounds could therefore be vital for the production of novel chemicals and second generation bioethanol.

Having seen that *Pseudomonas* and *Corynebacterium* possess potent oxidoreductases that efficiently convert eugenol via coniferyl aldehyde^11^,^12^,^13^, we speculated that similar enzymes could be present in *S. cerevisiae*. The main aim of this study was thus to identify endogenous enzymes that are actively involved in the conversion of CA in *S. cerevisiae.* Furthermore, we wanted to metabolically engineer *S. cerevisiae* to obtain a strain with increased capacity to catabolize and possibly, increased tolerance to CA. We searched for enzymes that may be involved in the aerobic conversion of CA in *S. cerevisiae.*

We have previously proposed a pathway for the conversion of CA, ferulic acid (FA) and p-coumaric acid (PCA)^5^, in the present study, a *S. cerevisiae* strain overexpressing the proposed pathway was developed. We selected Ald5 based on sequence similarity between *ALD5* gene in *S. cerevisiae* and *CALDH* gene from *Pseudomonas sp.* strain HR199^14^*. ALD5* is involved in the conversion of acetaldehyde to acetate^15^. *PAD1* has been reported to be involved in the conversion of cinnamic acids to styrene and alcohols^8^,^9^, we therefore overexpressed *PAD1* for the conversion of cinnamic acids derived from the conversion of CA. The conversion of phenolic alcohols and the relocation or cleavage of the methyl functional groups on the phenolic alcohols indicated the action of methyl transferases^16^. The alcohol acetyl transferases *ATF1* and *ATF2 are* known to be involved in the metabolism of various aromatic esters such as phenyl ethyl acetate and aliphatic esters in *S. cerevisiae.* Acetyl transferases are also known to be involved in the conversion of inhibitory compounds^16^,^17^,^18^. We hypothesized that overexpression of *ATF1* and *ATF2* would improve the conversion of phenolic alcohols formed as intermediates and facilitate the entire catabolic process of CA, FA and PCA. *ALD5, PAD1, ATF1* and *ATF2* were overexpressed on the hypothesis that their corresponding enzymes are active in our previously proposed conversion pathway^5^. This resulted in a new strain of *S. cerevisiae* named *APT_1.*

To better understand the metabolic consequences of the genetic changes, the strains was thoroughly physiologically characterized. This included mapping the phenolic metabolites and studying the effects of CA, FA and PCA on the physiology of the new *S. cerevisiae APT_1* strain. *APT_1* was compared to strains *SC_ald5Δ, B_CALD* which we have engineered to heterologously express coniferyl aldehyde dehydrogenase (*CALDH*) from *Pseudomonas sp.* strain HR199 and the control strain, all of which we have previously reported^14^.

## Results

The catabolism of CA was investigated in *APT_1*. This was compared to catabolism of CA previously reported for *B_CALD*, over-expressing *CALDH* from *Pseudomonas sp* HR199, an isogenic *S. cerevisiae* strain named *SC_ald5Δ* in which *ALD5* had been deleted and a control *S. cerevisiae* strain^14^. In addition, *in vitro* and *in vivo* assays were carried out on *APT_1, B_CALD, SC_ald5Δ* and control strains to test their ability to convert FA and PCA.

**Figure 1:**
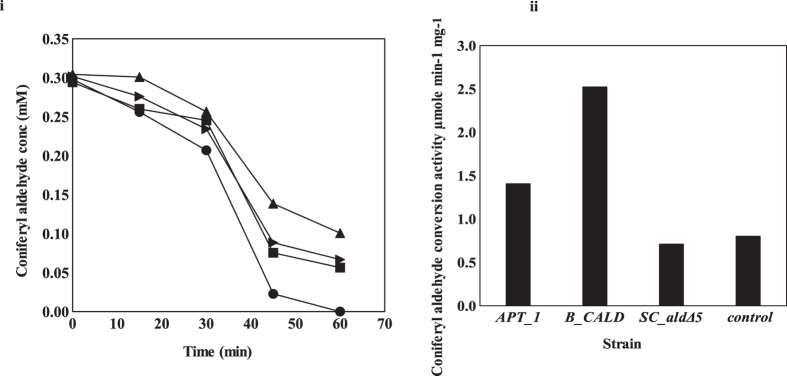
(i) *In vitro* analysis of the conversion of coniferyl aldehyde in *APT_1* (⚫), *B_CALD* (■), *SC_ald5Δ* (▲), and the control strain (▬). (ii) Coniferyl aldehyde conversion activity in *APT_1, B_CALD, SC_ald5Δ* and the control strain.

Acetaldehyde dehydrogenase assay on cell free extracts (CFE) from strain *APT_1* showed that the strain had a specific activity of 9.02 ± 0.10 mU mg^−1^. The activity was ∼1.9 times the activity of the parental control strain previously reported^14^ and ∼2.3 times the background activity in the *SC_ald5Δ* strain, also previously reported^14^. The *in vitro* conversion of CA with CFE revealed that *APT_1*, converted 0.5 mM of CA over a period of 60 minutes (Fig. 1)*. APT_1* exhibited an activity of 1.41 ± 0.05 μmole min^−1^ mg^−1^. This activity is ∼1.8 times that of the parental control strain and ∼2 times the background activity in the isogenic strain *SC_ald5Δ*, as previously reported^14^.

### Strain engineering of *APT_1* with *PAD1* confers higher ferulic acid conversion capacity

**Figure 2:**
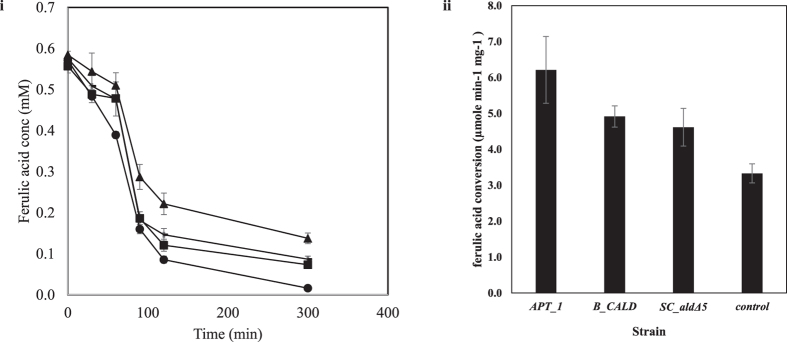
(i) *In vitro* analysis of ferulic acid conversion in *APT_1* (●), *B_CALD* (■), *SC_ald5Δ* (▲), and the control strain (▬). (ii) Ferulic acid conversion activity in *APT_1, B_CALD, SC_ald5Δ* and the control strain.

The GC-MS analysis showed that *APT_1* converted FA most rapidly (Fig. 2i). 0.6 mM of FA was converted over a period of 3 hours by the CFE from *APT_1*, with 85% of the conversion taking place in 2 hours. 0.6 mM of FA was not completely converted by CFE from *B_CALD, SC_ald5Δ* and the control strain, all without *PAD1* overexpression. *APT_1* CFE exhibited the highest activity at 6.21 ± 0.09 μmole min^−1^ mg^−1^ (Fig. 2ii).

### *In vitro* conversion of isoamyl alcohol in recombinant strains

**Figure 3:**
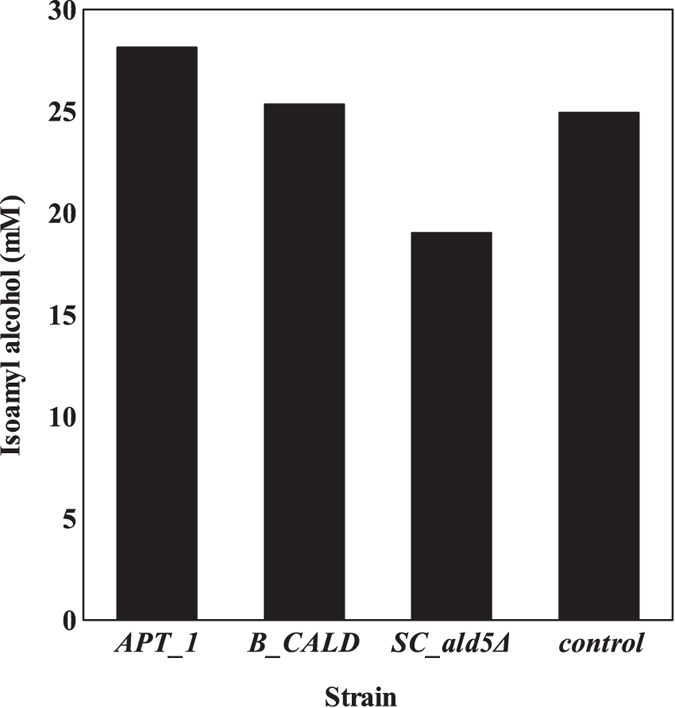
*In vitro* conversion of isoamyl alcohol by *APT_1, B_CALD, SC_ald5Δ*, and the control strain.

Increased conversion of isoamyl alcohol was expected to result from the overexpression of the alcohol acetyl transferase genes *ATF1* and *ATF2.* The GC-MS analysis of the *in vitro* conversion of isoamyl alcohol revealed that *APT_1*, converted the highest amount of isoamyl alcohol in 60 minutes (Fig. 3). 28.13 ± 0.40 mM of isoamyl alcohol was converted over a period of 60 minutes by the CFE from the *APT_1* strain, while CFE from *B_CALD, SC_ald5Δ* and the control strain, respectively converted 25.35 ± 1.4 mM, 19.02 ± 1.1 mM and 24.92 ± 1.3 mM, of isoamyl alcohol, respectively. The *SC_ald5Δ* strain exhibited the lowest conversion of isoamyl alcohol.

### Catabolism of coniferyl aldehyde, ferulic acid and p-coumaric acid

**Figure 4:**
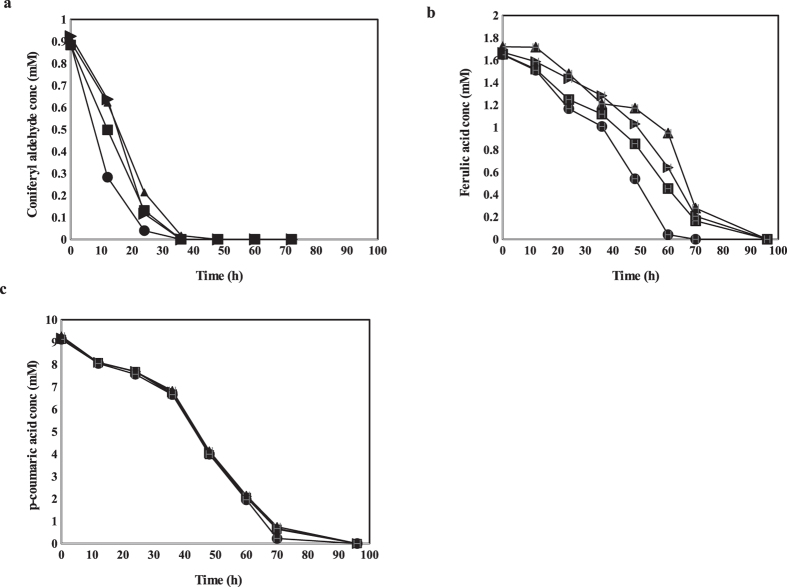
Conversion of (**a**) coniferyl aldehyde, (**b**) ferulic acid and (**c**) p-coumaric acid in the in *APT_1* (●), *B_CALD* (■), *SC_ald5Δ* (▲), and the control strain (▬).at 1.1 mM coniferyl aldehyde, 1.8 mM ferulic acid and 9.7 mM p-coumaric acid.

The *in vivo* conversion of CA, FA and PCA by strains *APT_1, B_CALD, SC_ald5Δ* and the control strain was monitored separately in identical, triplicate bioreactor cultivations. A transient conversion process in which intermediates were being formed and converted, was observed for the three compounds and their conversion products. A total conversion of 1.1 mM CA in the culture medium was observed in all the strains. The conversion occurred over a period of 36 hours in *APT_1, B_CALD* and the control strain while it lasted for 48 hours in strain *SC_ald5Δ* (Fig. 4a). During the first 24 hours of cultivation, the volumetric conversion rate of CA was similar in the *APT_1* and the *B_CALD* strains; 0.035 ± 0.005 mM h^−1^ and 0.034 ± 0.0005 mM h^−1^, respectively. The volumetric conversion rates in the *SC_ald5Δ* and the control strains were different; 0.029 ± 0.0005 mM h^−1^ and 0.031 ± 0.0005 mM h^−1^ respectively.

The conversion of FA was most rapid in the *APT_1* strain which converted all the FA in 60 hours while the other strains required 96 hours (Fig. 4b). Volumetric conversion rates of FA were significantly different among the strains (Table 1). In the first 60 hours of cultivation, the highest volumetric conversion rate of FA was observed in *APT_1* at 0.026 ± 0.001 mM h^−1^ and slowest in the *SC_ald5Δ* strain at 0.014 ± 0.001 mM h^−1^. Surprisingly, the volumetric conversion rate of PCA was similar among the four strains (Table 1), although the *APT_1* strain converted PCA slightly more rapidly, being complete after 72 hours (Fig. 4c).

**Figure 5:**
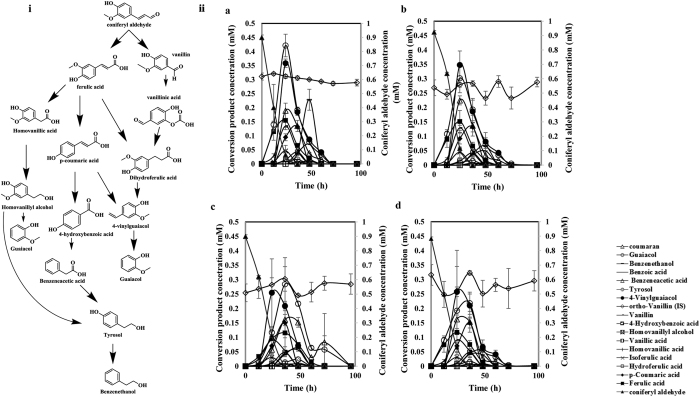
(i) Proposed conversion route of coniferyl aldehyde into other phenolic compounds. (ii) Conversion product profile of coniferyl aldehyde for (**a**) *APT_1*, (**b**) *B_CALD*, (**c**) *SC_ald5Δ*, and (**d**) the control strain.

A range of transient, conversion products was detected from the conversion of each of CA, FA and PCA. Each product appeared in the products pool at different times, increased to a peak concentration and declined until it became undetectable. The conversion products of the phenolic compounds were closely monitored and are arranged in the order in which they have appeared and transitioned in time in Figs 5, S3 and S4, Tables S1–S3. The conversion of CA yielded the highest number of intermediates. The product profiles gave insight into product formation pattern, starting with CA to benzenethanol, the last product detected at the end of cultivations (Fig. 5i). The conversion of FA differed slightly between the strains with respect to the time at which conversion products concentrations peaked (Figure S3). The most abundant products for *APT_1, B_CALD* and the control strains were benzenethanol, tyrosol, isoferulic acid, 4-vinylguaiacol and p-coumaric acid. The overall product profile was similar for all four strains, however, the product titres differed (Table S2). The overall PCA conversion product profile did not differ between the four strains (Figure S4i), however the metabolite levels and progression were different (Figure S4 ii, Table S3). The 5 most abundant products for all strains were guaiacol, benzenethanol, benzeneacetic acid, tyrosol, and 4-hydroxybenzoic acid.

The overall products profile for both FA and PCA revealed a conversion pattern that is very similar to that of CA (For details, see supplementary information. Also Figures S3–S4). For all the strains, the last remaining compound after 96 hours of cultivation were benzenethanol and guaiacol. The titres of the conversion products formed were proportional to the titres of the starting compounds with PCA conversion yielding the highest titres of products because the starting titre was highest (Figure S4 ii).

Conversion products of CA and their appearance throughout the cultivation time is described in detail in the following section. Conversion of FA and PCA followed a similar, yet not identical pattern and is described in supplementary text.

### Conversion products of coniferyl aldehyde

During the conversion of CA, FA was the first metabolite detected, within the first 2 hours of cultivation (data not shown). This is consistent with our previous findings^5^. The five most abundant compounds in the conversion of CA by *APT_1* strain were guaiacol, 4-vinylguaiacol, benzeneacetic acid, benzenethanol and ferulic acid (Figure S5). The product profile of *B_CALD* strain was similar to that of *APT_1* strain with guaiacol, 4-vinylguaiacol, benzeneacetic acid, FA and PCA being its most abundant compounds. Most cinnamic acids formed early in the conversion and peaked within the first 24 hours of cultivation. Phenolic alcohols also peaked within 24 hours. The highest concentrations of guaiacol (0.42 ± 0.04 mM) and 4-vinylguaiacol (0.36 ± 0.02 mM) during the cultivations were observed in *APT_1* strain (Fig. 5iia).

Conversion of CA was slowest in *SC_ald5Δ* strain, as a result intermediate products formation was also slow. The five most abundant intermediate products were 4-vinylguaiacol, benzeneacetic acid, benzenethanol, ferulic acid and vanillin. The most abundant intermediate product was guaiacol (0.28 ± 0.09 mM) at 36 hours while 4-vinylguaiacol which had earlier peaked at 24 hours was the second most abundant product (0.26 ± 0.12 mM) (Table S1). The cinnamic acids in the *SC_ald5Δ* strain peaked at 36 hours and their concentrations were lowest compared to other strains. For example, the ferulic acid concentration peaked at 0.11 ± 0.06 mM, compared to 0.15 ± 0.03 mM in the *APT_1* strain (Table S2). Although the CA conversion products peaked at different times and the titres varied among the four strains, however 4-vinylguaiacol consistently peaked at 24 hours in all the strains.

Complete conversion of CA was observed in the four strains. The CA conversion products were also observed to be absent from the cultivations at later times, suggesting that the conversion products were further and completely catabolized by the cells. Strain *SC_ald5Δ* exhibited the slowest conversion of CA and its products, requiring a period of 96 hours (Fig. 5iic, Table S1). The overall product formation profile was similar in all strains, leading to the suggested conversion profile for CA in Fig. 5i.

### Effect of conversion of coniferyl aldehyde, ferulic acid and p-coumaric acid on the physiology of recombinant strains

The physiological performances of the strains varied both in the YMMM control medium and when cultivated in the presence of the phenolic compounds. In YMMM, the maximum specific growth rates of all the strains were in the range of 0.36 ± 0.01 h^−1^ to 0.38 ± 0.03 h^−1^ (Table 2). As listed in Table 2, the *APT_1* strain exhibited the highest ethanol yield at 0.19 g g^−1^ in comparison with the *B_CALD, SC_ald5Δ* and control respectively having an ethanol yield of 0.14 g g^−1^, 0.14 g g^−1^and 0.17 g g^−1^. The *APT_1* also had the highest biomass yield at 0.34 g g^−1^ in comparison with the *B_CALD, SC_ald5Δ* and control respectively having 0.20 g g^−1^, 0.26 g g^−1^and 0.20 g g^−1^. Acetate yield was the lowest in *APT_1* at 0.003 g g^−1^ in comparison with the *B_CALD, SC_ald5Δ* and control respectively having 0.007 g g^−1^, 0.004 + 0.001 g g^−1^ and 0.007 + 0.001 g g^−1^. Glycerol yield was also very low in *APT_1* at 0.015 g g^−1^ lower than in the *B_CALD, SC_ald5Δ* strains and only 50% of that of the control (Table 2).

In the presence of 1.1 mM CA under aerobic batch cultivation condition, all the strains experienced a lag phase. While strains *APT_1, SC_ald5Δ* and the control experienced a lag phase of 14 hours, the *B_CALD* experienced a lag phase of 36 hours as previously reported^14^. The maximum specific growth rates of strains *APT_1* was 0.21 + 0.01 h^−1^ compared to that of the *B_CALD, SC_ald5Δ* and the control which we had previously reported to be 0.18 + 0.02 h^−1^, 0.24 + 0.05 h^−1^ and 0.24 + 0.01 h^−1^ respectively^14^. While ethanol and glycerol yields were not different between the strains, the biomass, acetate and CO_2_ yields varied (Table 2). The biomass, acetate and CO_2_ yields of *APT_1* were respectively 0.10 gg^−1^, 0.01 gg^−1^ and 0.71 gg^−1^. These were not different from those measured or previously reported for the *B_CALD, SC_ald5Δ* and control strains^14^ (also Table 2).

In the presence of 1.8 mM ferulic acid, the maximum specific growth rates of strains *APT_1* was 0.28 h^−1^ compared to that of the *B_CALD, SC_ald5Δ* and the control which were 0.25 h^−1^, 0.3 h^−1^ and 0.27 h^−1^ respectively (Table 2). Also, in the presence of 9.7 mM p-coumaric acid, the maximum specific growth rates of strains *APT_1* was 0.21 h^−1^ compared to that of the *B_CALD, SC_ald5Δ* and the control which were 0.23 h^−1^, 0.2 h^−1^ and 0.22 h^−1^ respectively. Surprisingly, the yields of the fermentation metabolites were not significantly different from each other in the presence of ferulic and p-coumaric acids.

### Resistance to coniferyl aldehyde

Resistance to coniferyl aldehyde was determined through a toxicity test using Bioscreen in which *APT_1, B_CALD, SC_ald5Δ* and the control strains were cultivated in the presence of different concentrations of coniferyl aldehyde. The resistance was assessed by the ability of the cells to grow in the presence of coniferyl aldehyde, and the calculation of their maximum specific growth rates were calculated. It was observed that *APT_1* showed the highest tolerance to coniferyl aldehyde, being the only strain that could grow in the presence of 1.4 mM coniferyl aldehyde (data not shown). As can be seen from Table 3, the maximum specific growth rate of the strains decreased with increasing concentration of coniferyl aldehyde. At 1.4 mM, the *APT_1* still exhibited a specific growth rate of 0.074 + 0.02 h^−1^ while no growth was observed in the other strains. A prolonged lag phase of 22 hours was observed in the *APT_1* in the presence of higher concentrations of coniferyl aldehyde (data not shown).

## Discussion

The overall aims of this study were: 1. to identify endogenous of *S. cerevisiae* enzymes that are actively involved in the conversion of phenolic compounds, especially coniferyl aldehyde (CA), ferulic acid (FA) and *p*-coumaric acid (PCA);.2. to engineer *S. cerevisiae* strain with increased tolerance to CA. The results from the experiments showed that *APT_1* strain had the best performance: converting of 1.1 mM coniferyl aldehyde in less than 36 hours, 1.8 mM ferulic acid and 9.7 mM p-coumaric acid both in 60 hours, and showing an ability to grow in the presence of 1.4 mM coniferyl aldehyde, while other strains showed no growth in the Bioscreen. The *SC_ald5Δ* strain on the contrary exhibited the poorest performance. This supports the hypothesis that *ALD5*, is actively involved in the conversion of, and resistance to phenolic compounds. The aldehyde dehydrogenase family in *S. cerevisiae* consists of 5 members, which have been characterized and sequentially named *ALD2-ALD6. ALD6* (YPL061w), *ALD2* (YMR170c) and *ALD3* (YMR169c) are cytosolic, while *ALD4* (YOR374w) and *ALD5* (YER073w) are mitochondrial^19^. The *ALD* genes have also been reported to exhibit redundancy, although they use different co-factors^19^,^20^. The redundancy in the *ALD* gene family may explain why the *SC_ald5Δ* strain was still capable of converting CA, despite the fact that it exhibited a higher sensitivity to coniferyl aldehyde, and the volumetric conversion rate was lower than in the other strains.

The inability of the *B_CALD* strain to outperform the *APT_1* strain in converting CA, despite the fact that it exhibited the highest acetaldehyde dehydrogenase activity, was unexpected, especially considering that its native *ALD5* had not been deleted and a synergistic effect was anticipated. One plausible explanation is that although the conversion of CA to cinnamic acids was efficient in the strain, there was a constraint downstream the conversion process, since other enzymes relevant for the entire conversion process were not over-expressed.

The complete conversion of CA and its conversion products observed in this study may be indicative of the ability of *S. cerevisiae* to utilise phenolic compounds. It is important to note that the conversion of CA may have been divided between three main paths leading to two products; guaiacol and benzenethanol. The first route being coniferyl aldehyde→vanillin→vanillic acid→dihydroferulic acid→4vinyl guaiacol→guaiacol. The second possible path is coniferyl aldehyde-→ferulic acid→homovanillic acid→homovanillyl alcohol→guaiacol. The third possible route is coniferyl aldehyde→ferulic acid→p-coumaric acid→4hydroxybenzoic acid→benzeneacetic acid→tyrosol→benzenethanol. Based on the structure of the conversion products and their time of formation, it is proposed that these three possible conversion routes were active simultaneously.

The conversion of CA was directed towards guaiacol and benzenethanol, which the cells may metabolize. It is known that *S. cerevisiae* and several other microorganisms can degrade catechol^21^ through the β-ketoadipate pathway^21^,^22^. It has also been shown that *S. cerevisiae* cannot degrade 4-hydroxybenzoate due to the lack of benzoate-4-hydroxylase activity^23^,^24^. Guaiacol is a catechol monomethyl ether, with a hydroxyl group at the C1 and a methoxy group at the C2 position. Our hypothesis is that the *S. cerevisiae* metabolizes CA via guaiacol by hydrolysing the methoxy group on guaiacol into a hydroxyl group to form catechol. Via the _β_-ketoadipate pathway, the bonds between the two carbon atoms bearing the hydroxyl groups in catechol is broken to open up the aryl ring for subsequent assimilation. In support of this hypothesis we found that guaiacol was consistently the most abundant metabolite formed in the *APT_1* strain, which may be explain the rapid conversion of CA and all its products in the *APT_*1 strain. Furthermore, this may be the reason why the *APT_1* strain, also consistently converted all the FA and PCA and most of their conversion compounds faster than the other strains investigated in this study. In our previous study^5^, we did a toxicity screening on the conversion products of CA, FA and PCA. The products tested included benzenethanol. We observed that the growth of *S. cerevisiae* did not cease even in the presence of 22.1 mM benzenethanol (Phenyl ethyl alcohol). Based on our increased understanding of the catabolism of phenolic compounds in this current study, we believe benzenethanol did not arrest the growth of *S. cerevisiae* because it was being utilised by the cells.

The similarity in the conversion product profiles for CA, FA and PCA supports the conversion route earlier hypothesized for the three compounds in *S. cerevisiae*. The most rapid conversion of FA and PCA observed in *APT_1* cultivations indicates that *APT_1* was enhanced by the overexpression of *PAD1* which has been reported to be involved in the conversion of ferulic acid to styrene and 4-vinyl guaiacol^8^. Several products derived from the conversion of CA, FA and PCA in this study have not been previously reported. The formation of other phenolic aldehydes such as vanillin, several phenolic acids such as vanillic and homovanillic acids and the probable simultaneous conversion of the phenolic compounds through multiple route, may be an indication of the activity of some other enzymes yet unidentified but crucially involved in phenolics catabolism in *S. cerevisiae*.

The persistently high level of benzenethanol is an indication of the increased activity of alcohol acetyl transferases in the *APT_1* strain. Furthermore, the formation of benzenethanol appeared to be the longest conversion route for the phenolic compounds and was apparently favoured by the lack of methoxy group on compounds such as PCA and other conversion products similarly lacking a methoxy group. The methoxy group seem to favour the conversion of phenolic compounds via guaiacol rather than via benzenethanol. For instance, in the conversion of p-coumaric acid, significant amount of tyrosol was observed to be formed, further converted to benzenethanol, rather than via the guaiacol route.

The conversion of isoamyl alcohol to isoamyl acetate is an esterification reaction that has been shown to be performed by alcohol acetyl transferases in *S. cerevisiae*^25^,^26^. Also, *ALD5* has been shown to be involved in the conversion of fusel aldehydes to fusel acid via a reduction process in the Ehrlich pathway^27^. Oxidation of fusel aldehyde in the Ehrlich pathway forms fusel alcohol, one of which is isoamyl alcohol. Although this was not considered at the time of strain construction, the deletion of *ALD5* might have influenced how the esterification of isoamyl alcohol to isoamyl acetate is effectively carried out, this could have been the reason for the reduced isoamyl conversion observed in the *SC_ald5Δ* strain.

Overall, the *APT_1* strain exhibited efficient and rapid conversion of CA, FA and PCA, traits that are valuable when developing microorganisms that are both robust and useful for a more efficient utilization of lignocellulosic substrates and production of specific metabolites. This give the *APT_1* an advantage and demonstrates our strategy of strain development has been successful.

## Conclusion

We have demonstrated that *ALD5* is actively involved in the conversion of coniferyl aldehyde in *S. cerevisiae* in *APT_1. APT_1* also exhibited improved capability to convert coniferyl aldehyde, ferulic acid and p-coumaric and increased resistance to coniferyl aldehyde. This showed that combined overexpression of *ALD5, PAD1, ATF1* and *ATF2* helps *S. cerevisiae* in phenolics conversion and tolerance. Consequently, the *APT_1* strain is a strain that has potential for use in biorefinery applications due to its ability to both catabolize phenolic compounds and produce ethanol. It is important that the individual roles of these genes and their corresponding enzymes be further investigated in order to elucidate their specific roles in phenolics conversion in *S. cerevisiae*, in greater details. Of particular importance is the demethoxylation of phenolic compounds which we hypothesized is performed by the *ATF1* and *ATF2.*

The phenolics conversion demonstrated in this study, demonstrates the not so obvious ability of *S. cerevisiae* to catabolise various phenolic compounds. Thus, the endogenous genes of *S. cerevisiae* can be further investigated and enhanced for bioconversion of phenolic compounds. The conversion route of phenolic compounds present us with a better understanding of the conversion process phenolic compounds go through and offers an insight into products which otherwise inhibitory compounds can be converted into using *S. cerevisiae*.

## Materials and Methods

### Materials

#### Saccharomyces cerevisiae strain

*Saccharomyces cerevisiae* CEN.PK 102-3A strain was the parental strain of the *APT_1* strain created and used for the present study. We also used strains, *B_CALD, SC_ald5Δ* and the control which we have constructed and reported in a different study^14^. The other *Saccharomyces cerevisiae* strains; *SC_ald5Δ* and the control also originated from the parental strain CEN.PK 102-3A while *B_CALD* was developed from strain CEN.PK. 113-7D. The strains and their genotypic information used in the study are compiled in Table 4.

#### Escherichia coli

NEB 5-alpha strain of competent *E. coli* cells was used for plasmid construction in this study. The competent cells were developed by New England Biolabs Inc. and were obtained alongside Gibson assembly kit from BioNordika, Sweden.

### Strains and plasmids construction

The background plasmids used in this study are YIplac211 and YIplac128^28^, (Table 4). *TDH3* and *CYCt* were both amplified from digested plasmid DNA. *TDH3* was amplified with the following primers TDH3_fwd AAGCTTCAGTTCGAGTTTATCATT, TDH3_rev CTGCAGGTGTGTTTATTCGAAAC while *CYCt* was amplified with primer set; CYCter_fwd GGATCCCCGGGTACCGA and CYCter_rev GAATTCGCAAATTAAAGCCTTCGAG. *ALD5, PAD1, ATF1* and *ATF2* were amplified from *S. cerevisiae* genomic DNA. *ALD5* was amplified with the following primer set; ALD5_fwd ATGCTTTCTCGCACAAGAGCT and ALD5_rev TCAACGAATTGGCTTGTCAATG. *PAD1* was amplified with the following primer set; PAD1_fwd ATGCTCCTATTTCCAAGAAGA and PAD1_rev TTACTTGCTTTTTATTCCTTCC. *ATF1* was amplified using the primer set; ATF1_fwd ATGAATGAAATCGATGAGA and ATF1_rev CTAAGGGCCTAAAAGGAGAGCTTT while ATF2 was amplified using the primer set ATF2_fwd ATGGAAGATATAGAAGGATACGAACC.

ATF2_rev TTAAAGCGACGCAAATTCG. The PCR products were cloned into the YIp plasmids. All plasmid cloning was done using the Gibson DNA assembly protocol^29^ (Figure S1). *TDH3-ALD5-CYCt TDH3-PAD1-CYCt* was cloned into the YIplac128 plasmid while *TDH3-ATF1-CYCt TDH3-ATF2-CYCt* was cloned into YIp211 plasmid (Figure S2). The primer information for the construction of plasmids is supplied in supplementary information S1. DNA sequences of constructed hybrid plasmids were confirmed by DNA sequencing (Eurofins MWG). The Yip LEU ALD5PAD1 and Yip URA ATF1ATF2 were transformed into the *CEN.PK 102-*3A strain to construct the *APT_1* strain. The construction of *B_CALD* strain using synthetic, codon optimized *CALDH* obtained from Genescript has been previously reported^14^. We also previously reported the construction of the control strain and the deletion of *ALD5* in the *CEN.PK 102-3A* to create the *SC_ald5Δ* strain^14^.

### Protein sequence accession numbers

The nucleotides sequences accession numbers of the *ALD5, PAD1, ATF1, ATF2* and *CALDH* respectively are NP_010996, DAA12367, CAA99708, CAA97203 and WP_016502080 respectively.

### Selection criteria for genes of interest

As, we have previously proposed a pathway for the conversion of coniferyl aldehyde, ferulic acid and p-coumaric acid^5^, we prospected for an aldehyde dehydrogenase in *S. cerevisiae*. After multiple comparisons, Ald5 was selected as the most probably enzyme candidate oxidizing coniferyl aldehyde in yeast, based on sequence similarity between *ALD5* gene in *S. cerevisiae* and *CALDH* gene from *Pseudomonas sp.* strain HR199^14^. The National Center for Biotechnology Information Basic Local Alignment Search Tool (BLAST) and the conserved domain data base on NCBI were used^31^. *ALD5* is located in the mitochondrion, it belongs to the aldehyde dehydrogenase family in *Saccharomyces cerevisiae*^15^. Pad1 was selected based on its reported roles in literature^8^,^9^. Atf1 and Atf2 were selected based on their known roles in *S. cerevisiae*^16^,^17^,^18^.

### Chemicals

All chemicals used in the cultivation medium were obtained from Sigma-Aldrich, Germany. Unless otherwise stated, coniferyl aldehyde, ferulic and p-coumaric acids and all the other phenolic compounds used as standards for analytical method development were procured from Sigma-Aldrich GmbH, Germany. All the chemicals used in the GC-MS analyses were PA graded chemicals. Acetone, ethyl acetate and dichloromethane were procured from Merck, Germany. N,O-bis(trimethylsilyl) trifluoroacetamide (BSTFA) and 2,6-diethylnaphtalene were procured from Sigma-Aldrich, Germany. O-vanillin, the internal standard used during GC-MS analyses was procured from Fluka, Sweden.

## Methods

### Preparation of cultivation medium

The basal medium used for all cultivations was the yeast minimal mineral medium (YMMM)^32^. The phenolics medium were separately prepared as YMMM containing 1.1 mM coniferyl aldehyde (1.1 mM coniferyl aldehyde in YMMM) or YMMM containing 1.8 mM ferulic acid or YMMM containing 9.7 mM of p-coumaric acid. The concentrations of coniferyl aldehyde, ferulic and p-coumaric acids used in each medium, had all been previously determined in a toxicity screening experiment where increasing concentration of the respective phenolic compound was used^33^.

### Cultivation of *Escherichia coli*

The *E. coli* was cultivated in lysogeny broth medium [LB]^34^ at 37 °C in baffled Erlenmeyer flasks on a rotary shaker at 180 rpm.

### Cultivation of *Saccharomyces cerevisiae strains*

The inoculum was cultivated in YMMM in 500 ml Erlenmeyer flasks, incubated at 30 °C and shaking on a rotary shaker at 200 rpm for a period of 18 hours in YMMM. Cells were harvested from a volume of inoculum corresponding to an OD_600_ of 0.2 in a 700 ml culture by centrifugation at 3000 rpm for 5 minutes at room temperature. The cell were resuspended in fresh cultivation medium and immediately used to inoculate the main cultivation. The main cultivations were carried out in DASGIP parallel bioreactor systems consisting of two units, each consisting of four SR0700ODLS vessels (DASGIP, Jülich, Germany). Aeration was set to 1 vvm. The culture volume was 700 ml and the fermenters were preconditioned to the culture condition overnight at pH 5. Cultivations were run for 96 hours with aeration. A feedback loop was created between the signal from the dissolved oxygen probe signal and the impeller speed to maintain aeration above 40% of oxygen saturation. Triplicate cultivations were performed for each strain and set of conditions.

### Optical density measurement of culture for growth determination

Growth was monitored by measurement of the optical density of the culture at 600 nm (OD_600_) using a Thermo Scientific GENESYS 20 Visible Spectrophotometer.

### Determination of dry cell weight

Dry cell weight was determined in triplicate. Cells were harvested via filtration, from a 5 ml sample of each culture, using pre-dried and pre-weighed filter paper discs of 0.45 μm pore size. Filtration was performed using a water tap vacuum filter unit (both from Sartorius Stedim Biotech, Göttingen, Germany). The samples and the filter paper discs were dried in a microwave oven at 120 W for 15 minutes, cooled in a desiccator and weighed again. The biomass was determined from the weight difference.

### Determination of maximum specific growth rates

The maximum specific growth rates were determined by plotting the natural logarithm of OD600 of the culture samples against the cultivation time. The maximum specific growth rates calculated from the readings obtained from the Bioscreen (OD_Bioscreen_) were transformed into standard spectrophotometric values (OD_spectro_) at OD_600_, using the relation:


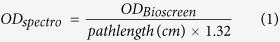


where: OD_spectro_ is the equivalent OD on spectrophotometer at 600 nm and OD_Bioscreen_ is the OD measured on the Bioscreen


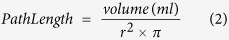


where: *volume* is the culture volume in a growth chamber in the Bioscreen plate and *r* is the radius of the chamber.

Non-linearity at higher cell densities was corrected as described by Warringer *et al*.^35^ using the expression:


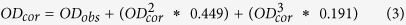


where: OD_cor_ is the corrected OD and OD_obs_ is the observed OD values, from which the average blank value has been subtracted.

### Determination of yields and rates

Ethanol, glycerol, acetate, carbon dioxide and biomass yields from glucose were calculated during the exponential growth phase from the plot of titres of each of each products against the glucose consumed. The yield for each product was obtained from the slope of a linear regression fit to the plot. Average values of biological replicates were used as the final yield for each culture condition.

The specific consumption rate of the substrate (glucose) was calculated using the relation:


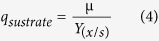


where q_*substrate*_ is the specific substrate consumption rate, μ the maximum specific growth rate, and Y_(x/s)_ the biomass yield coefficient.

The specific productivity rates of ethanol, biomass, glycerol and acetate were determined using the relation:


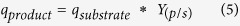


where Y_(p/s)_ is the product yield coefficient.

During the respiratory growth phase, the biomass yield Y_(x/s)_, was determined using a combination of glycerol, acetate and ethanol as substrate. The average rate of conversion of coniferyl aldehyde was determined as the slope of the plot of the titres of coniferyl aldehyde against time. The conversion rates were determined during the period of cultivation in which the phenolic compounds being converted was present in all four cultivations.

### Analytical methods

#### Analysis of metabolites

The fermentation metabolites from the cultivation were analyzed using high performance liquid chromatography (HPLC). A Dionex Ultimate 3000 HPLC unit (Thermo Scientific, Dionex Corporation, Sunnyvale, USA) equipped with an Aminex HPX-87H (Bio-Rad, USA) column was used for the analyses. The column temperature was set to 45 °C, and 5 mM H_2_SO_4_ was used as the mobile phase at a flow rate of 0.6 ml/min. Metabolites were quantified using a Shodex RI-101 refractive index detector and an Ultimate 3000 VWD 3100 variable wavelength ultraviolet detector coupled to the HPLC unit.

#### Time-based monitoring of the catabolism of coniferyl aldehyde, ferulic acid and p-coumaric acid

A 5 ml sample of the culture was rapidly collected, centrifuged at 5100 rpm at 0 °C for 5 minutes and the supernatants were stored at −20 °C until analyzed on gas chromatography–mass spectrometry (GC-MS). Prior to GC-MS analysis, a liquid-liquid extraction was carried out using 1 ml of the sample extracted with 1 ml ethyl acetate. Sample extraction was carried out at pH 2 in glass sample vials. O-vanillin (prepared in ethyl acetate) was added to each sample as the internal standard, at a final concentration of 50 μg/ml. The samples were vortexed on a multi-tube vortex at 2000 rpm for 20 minutes, thereafter allowed to stand for 10 min in order to have a proper phase separation between the solvent and water. For derivatization, 125 μl of the solvent phase (ethyl acetate) from the extracted sample was pipetted into a GC-MS vial and 87.5 μl derivatization reagent mix comprising of 12.5 μL Pyridine, 0.75 μL Trimethylchlorosilane (TMCS) and 74.25 μL of N,O-bis(trimethylsilyl)trifluoroacetamide (BSTFA) was rapidly added to each sample. The samples were capped and incubated in a rotary water bath at 70 °C and 120 rpm for 30 min. The GC-MS analysis was performed using DSQ II Single Quadrupole GC/MS chromatograph (Thermo Scientific, Germany). One μl of each sample was injected in a splitless mode, and the injector temperature was maintained at 250 °C. Separation was carried out on a DB-5 capillary column (Agilent, Sweden) with a length of 30 m, inner diameter of 0.32 mm and film thickness of 0.25 μm. Helium was used as the mobile phase at a flow rate of 1 ml/min. The temperature program was: 50 °C for 1 min, 5 °C/min to 350 °C, and then 350 °C for 5 min. Electron impact (EI+) was used for ionization in the mass spectrometer. Mass spectra were recorded from m/z 40 to 400 with a total cycle time of 0.7 s. The compounds with the highest abundance were identified by comparing the mass spectra with a NIST MS Search 2.0 library. Internal and external standards were used to determine the concentrations of the identified compounds.

### *In vitro* analysis for acetaldehyde dehydrogenase activity in cell free extract

Acetaldehyde dehydrogenase activity was determined as described previously^14^ using the method of Postma *et al*.^36^. Absorbance was measured at 340 nm using a FLUOstar Omega microplate reader (BMG LABTECH, Germany).

#### *In vitro* analysis of coniferyl aldehyde conversion in cell free extracts

*In vitro* analysis of coniferyl aldehyde conversion in cell free extracts was determined as previously reported^14^.

#### *In vitro* analysis of ferulic acid conversion using cell free extract

The conversion of ferulic acid was determined in all *S. cerevisiae* strains used in the study in order to confirm the overexpression of *PAD1* in the *APT_1* strain. The *S. cerevisiae* strains were cultivated to mid-exponential phase with an OD600 of 3.0. Cells were harvested from a 5-ml sample of culture by centrifugation at 4000 rpm for 5 minutes. The supernatant was discarded and the cell pellet was washed by resuspension in 5 ml ice-cold 100 mM sodium phosphate buffer, pH 6.0, and vortexing. The cells were again separated by centrifugation at 4000 rpm and resuspended in 1 ml ice cold 100 mM sodium phosphate containing 1× concentration of protease inhibitor. The suspension was transferred to 2 ml Eppendorf tube, containing acid washed glass beads and the cells were disrupted in a homogenizer running 2 disruption cycles each at 6ms^−1^ for 20 seconds and cooling on ice in between the disruption cycles. The suspended lysed cell mass and glass beads were separated by centrifugation at 1000 rpm for 5 minutes. The supernatant was transferred to a clean tube and immediately analyzed for Pad1 activity by incubating 800 μl of cell free extract with 200 μl of 3 mM ferulic acid to the extract, giving a final substrate concentration of 0.6 mM ferulic acid. The reaction mixture was incubated in a thermomixer at 30 °C and 150 μl of sample was withdrawn every 30 minutes for a total period of 5 hours. Withdrawn samples were immediately pipette into 1.5 ml Eppendorf tube heated to 95 °C in a thermomixer to stop reaction. After one minute heat inactivation, the sample was cooled down on ice and transferred into a glass vial holding 150 μl of ethyl acetate. The sample was extracted by shaking on a vortexer for 15 minutes. The ethyl acetate solvent fraction was carefully pipetted out into a fresh GC-MS tube, derivatised using TMCS, BSTFA and pyridine and analyzed on the GC-MS.

#### *In vitro* analysis of isoamyl alcohol conversion in strains

The conversion of isoamyl alcohol was determined in order to confirm the overexpression of *ATF1* and *ATF2*. A 5 ml sample of *S. cerevisiae* culture broth was collected and cooled on ice at 4 °C. The cells were harvested by centrifugation at 4000 rpm for 5 minutes at 4 °C. The cells were then resuspended and washed once with 5 ml of sterile distilled water, centrifuged and the supernatant was discarded. The cell pellet was washed by resuspension in 5 ml ice-cold washing buffer solution comprising 25 mM imidazole-HCl, 0.1 M NaCl, 20% glycerol, 1 mM dithiothreitol and 0.1% Triton X-100 at pH 7. The cells were again re-harvested by centrifugation at 4000 rpm and resuspended in 1 ml ice cold extraction buffer solution containing 25 mM imidazole-HCl, 0.1 M NaCl, 20% glycerol, 1 mM dithiothreitol, 0.1% Triton X-100 and 1× concentration of protease inhibitor at pH 7. The suspension was transferred to 2 ml Eppendorf tube holding acid washed glass beads and disrupted in a homogenizer running 2 disruption cycles each at 6 ms^−1^ for 20 seconds and cooling on ice in between. The suspended lysed cell mass and glass beads were separated by centrifugation at 1000 rpm for 5 minutes at 0 °C. 500 μl of the supernatant was transferred into a GC-MS glass sample vials and a final concentration of 0.5% isoamyl alcohol was added to start the alcohol acetyl transferase assay. The glass vials were tightly capped with silicone caps and incubated at room temperature and shaken for one hour. The vials were cooled on ice for 10 minutes and 500 μl of ethyl acetate was added, the samples were tightly capped again and extraction of isoamyl acetate was performed using a vortexer at 4 °C for 30 minutes. The ethyl acetate fraction of the samples were transferred to new vials, derivatised and analysed immediately for *ATF* activity by the residual amount of isoamyl alcohol detected and the amount of isoamyl acetate formed.

### Statistical validation of experimental data

All experimental data obtained were subjected to the student’s t-test to determine whether the differences between the results obtained with the difference strains were statistically significant. The number of replicates varied from 3 to 5, depending on the experiment. A t-test for two-sample assuming unequal variances was therefore performed. The significance level of probability was set at p < 0.05. All error bars are standard deviations from the averages of triplicate measurements of each parameter among biological replicates.

## Additional Information

**How to cite this article**: Adeboye, P. T. *et al*. *ALD5, PAD1, ATF1* and *ATF2* facilitate the catabolism of coniferyl aldehyde, ferulic acid and p-coumaric acid in *Saccharomyces cerevisiae. Sci. Rep.* **7**, 42635; doi: 10.1038/srep42635 (2017).

**Publisher's note:** Springer Nature remains neutral with regard to jurisdictional claims in published maps and institutional affiliations.

## Supplementary Material

Supplementary Information

## Figures and Tables

**Table 1:** Average volumetric rate of conversion of coniferyl aldehyde, ferulic acid and p-coumaric acid by *APT_1, B_CALD, SC_ald5Δ* and the control strains.

Strain	Conversion rate (mM h^−1^)		
	coniferyl aldehyde at 24 hrs	ferulic acid at 60 hr	p-coumaric acid at 72 hr
*APT_1*	0.035 ± 0.001	0.026 ± 0.001	0.128 ± 0.004
*B_CALD*	0.034 ± 0.001	0.017 ± 0.001	0.124 ± 0.004
*SC_aldΔ5*	0.029 ± 0.001	0.014 ± 0.001	0.124 ± 0.005
*Control*	0.031 ± 0.001	0.020 ± 0.001	0.121 ± 0.007

**Table 2:** Physiological influence of coniferyl aldehyde, ferulic acid and p-coumaric acid on the yield of fermentation metabolites on glucose by the strains *APT_1, B_CALD, SC_ald5Δ* and CTRL.

	μ max (h^−1^)	Yetoh/glu (g/g)	Yx/glu (g/g)	Yace/glu (g/g)	Ygly/glu (g/g)	YCO2/glu (g/g)
YMMM control						
* APT_1*	0.36 + 0.01	0.192 + 0.01	0.343 + 0.02	0.003 + 0.0002	0.015 + 0.001	0.38 + 0.02
* B_CALD*	0.37 + 0.02	0.138 + 0.001	0.201 + 0.01	0.007 + 0.0005	0.032 + 0.003	0.46 + 0.04
* SC_ald5Δ*	0.36 + 0.02	0.140 + 0.02	0.265 + 0.02	0.004 + 0.0014	0.020 + 0.001	0.41 + 0.002
* CTRL*	0.38 + 0.03	0.168 + 0.01	0.201 + 0.01	0.007 + 0.001	0.026 + 0.001	0.42 + 0.01
1.1 mM Coniferyl aldehyde						
* APT_1*	0.21 + 0.005	0.180 + 0.02	0.103 + 0.005	0.007 + 0.001	0.048 + 0.01	0.71 + 0.03
* B_CALD*	0.18 + 0.02	0.171 + 0.02	0.139 + 0.02	0.005 + 0. 001	0.049 + 0.003	0.83 + 0.08
* SC_ald5Δ*	0.24 + 0.04	0.181 + 0.02	0.110 + 0.01	0.009 + 0. 001	0.038 + 0.01	0.70 + 0.02
* CTRL*	0.26 + 0.01	0.184 + 0.01	0.112 + 0.01	0.010 + 0. 001	0.035 + 0.005	0.75 + 0.02
1.8 mM Ferulic acid						
* APT_1*	0.28 + 0.04	0.203 + 0.03	0.112 + 0.01	0.007 + 0.001	0.028 + 0.003	0.77 + 0.05
* B_CALD*	0.25 + 0.04	0.180 + 0.02	0.119 + 0.02	0.010 + 0.001	0.027 + 0.003	0.69 + 0.02
* SC_ald5Δ*	0.30 + 0.05	0.211 + 0.03	0.130 + 0.01	0.008 + 0.001	0.028 + 0.002	0.68 + 0.05
* CTRL*	0.27 + 0.07	0.197 + 0.03	0.126 + 0.02	0.009 + 0.001	0.028 + 0.003	0.59 + 0.07
9.7 mM p-coumaric acid						
* APT_1*	0.21 + 0.05	0.137 + 0.005	0.093 + 0.005	0.004 + 0.0001	0.036 + 0.004	0.85 + 0.05
* B_CALD*	0.23 + 0.02	0.159 + 0.001	0.082 + 0.002	0.003 + 0.0004	0.040 + 0. 004	0.89 + 0.02
* SC_ald5Δ*	0.24 + 0.01	0.157 + 0.002	0.097 + 0.005	0.004 + 0.0005	0.037 + 0. 003	0.95 + 0.07
* CTRL*	0.22 + 0.02	0.143 + 0.011	0.090 + 0.008	0.004 + 0.0004	0.040 + 0. 004	0.84 + 0.04

**Table 3:** Maximum specific growth rates (h^−1^) of *APT_1, B_CALD, SC_ald5Δ* and control strains at different concentration of Coniferyl aldehyde.

Strain	Maximum specific growth rates (h^−1^) at different concentration of Coniferyl aldehyde					
	Blank medium	0.67 mM	0.84 mM	1.01 mM	1.18 mM	1.4 mM
*APT_1*	0.28 + 0.02	0.20 + 0.03	0.18 + 0.03	0.12 + 0.03	0.10 + 0.01	0.07 + 0.02
*B_CALD*	0.27 + 0.01	0.13 + 0.04	0.15 + 0.03	0.12 + 0.02	0.08 + 0.01	—
*SC_ald5Δ*	0.28 + 0.02	0.22 + 0.04	0.13 + 0.02	0.06 + 0.01	0.09 + 0.01	—
*Control*	0.29 + 0.02	0.21 + 0.03	0.21 + 0.01	0.10 + 0.05	0.10 + 0.03	—

**Table 4:** Plasmids, *Escherichia coli* and *Saccharomyces cerevisiae* Strains used and constructed in the study.

*Escherichia coli* background strain	Recombinant strain	Genotype	source and reference
NEB 5-alpha Competent *E. coli*			New England Biolabs inc.
***Saccromyces cerevisiae*** **background strain**	**Recombinant strain**	**Genotype**	**source and reference**
CEN.PK 102-3A		MATa, leu, ura	30
CEN.PK. 113-7D		*MATa, MAL2-8 c, SUC2*	30
CEN.PK 102-3A	*APT_1*	*MATa, LEU, URA, ALD5, PAD1, ATF1* and *ATF2*	This study
CEN.PK. 113-7D	*B_CALD*	*MATa, MAL2-8 c, SUC2, CALDH*	14
CEN.PK 102-3A	*SC_ald5Δ*	*MATa, LEU, URA, ald5*	14
CEN.PK 102-3A	*control*	*MATa, LEU, URA*	14
**Native plasmid**	**Modified plasmid**	**character**	**source and reference**
YIplac211		*LEU*	28
YIplac128		*URA*	28
YIplac128	YIp LEU ALD5PAD1	*AmpR, LEU, TDH3p-ALD5-CYCt1, TDH3p-PAD1-CYCt1*	This study
YIplac211	YIp URA ATF1ATF2	*AmpR, URA, TDH3p-ATF1-CYCt1, TDH3p-ATF2-CYCt1*	This study
