# Supplementary material for: ALD5, PAD1, ATF1 and ATF2 facilitate the catabolism of coniferyl aldehyde, ferulic acid and p-coumaric acid in Saccharomyces cerevisiae
Source: Sci Rep. 2017 Feb 16;7:42635. doi: 10.1038/srep42635 (PMC5311992; doi:10.1038/srep42635)
Supplement: Supplementary Information [file srep42635-s1.pdf]

ALD5, PAD1, ATF1 and ATF2 facilitate the catabolism of coniferyl aldehyde, ferulic acid and p-coumaric acid in *Saccharomyces cerevisiae*

Peter Temitope Adeboye[a], Maurizio Bettiga[a] and Lisbeth Olsson[a,*].

[a.] Department of Biology and Biological Engineering, Division of Industrial Biotechnology, Chalmers University of Technology, SE-412 96 Gothenburg, Sweden

E-mail addresses

Peter Temitope Adeboye: adeboye@chalmers.se

Maurizio Bettiga: maurizio.bettiga@chalmers.se

Lisbeth Olsson: lisbeth.olsson@chalmers.se

Supplementary Information

Conversion products of ferulic acid

The 5 most abundant FA conversion intermediates in *APT_1*, *B_CALD* and the control strains were similar in concentrations, except for benzenethanol in *APT_1*. With a peak concentration of 0.50 ± 0.09 mM, this was higher than in the other strains. The 5 most abundant FA conversion products in *SC_ald5Δ* strain were different, they are; 4-Hydroxybenzoic acid, guaiacol, benzenethanol, 4-vinylguaiacol, and isoferulic acid were. Biggest differences were seen in the titres of 4-hydroxybenzoic acid and guaiacol. 4-hydroxybenzoic acid in the *APT_1, B_CALD* and the control strains were 0.030 ± 0.003 mM, 0.05 ± 0.03 mM and 0.06 ± 0.03 mM, respectively, while it was 0.17 ± 0.06 mM in the *SC_ald5Δ* strain (Figure S3 ii). Also, the peak concentrations of guaicol in the *APT_1,*

[*] Corresponding author

B_CALD and the control strains were 0.05 ± 0.01 mM, 0.05 ± 0.01 mM and 0.06 ± 0.00 mM respectively while it was 0.29 ± 0.06 mM in the *SC_ald5Δ* strain. The higher concentration of 4-hydroxybenzoic acid and guaiacol measured in the *SC_ald5Δ* strain cultivation was surprising because the deletion of *ALD5* was not expected to influence the conversion of cinnamic acids.

Conversion products of p-coumaric acid

The overall PCA conversion product profile did not differ between the four strains, however the metabolite levels and the time reached were different (Table S3). The 5 most abundant products for all strains were guaiacol, benzenethanol, benzeneacetic acid, tyrosol, and 4-hydroxybenzoic acid. The overall metabolite profile revealed a conversion pattern very similar to that of CA (Figure S4i). Benzenethanol was the only compound remaining after 96 hours of cultivation with all the strains. It was observed that the peak concentration of the most abundant PCA conversion products varied between the strains. In *APT_1*, the most abundant compounds were guaiacol, which reached a concentration of 1.70 ± 0.23 mM at 24 hours, and benzenethanol, which reached 1.69 ± 0.6 mM at 60 hours. With *B_CALD* strain, the highest titres were recorded for guaiacol, 1.53 ± 0.1 mM at 24 hours, and for tyrosol, 1.34 ± 0.14 mM at 24 hours. In *SC_ald5Δ* strain, the highest titres were recorded for tyrosol, 1.25 ± 0.15 mM at 24 hours, and for benzeneacetic acid, 1.103 ± 0.5 mM at 36 hours. Finally, in the case of the control strain, tyrosol peaked at 24 hours, 1.31 ± 0.18 mM and benzeneacetic acid at 36 hours, 1.25 ± 0.5 mM. The titre of tyrosol during the conversion of PCA to benzenethanol via tyrosol was significantly higher than that of other intermediates, supporting the hypothesis that PCA is mainly converted to benzenethanol (Table S3, Figure S4i).

Supplementary figure Legends

Figure S1. **i** Outline of plasmid assembled via Gibson protocol. **ii**. The principle of Gibson assembly for assembling DNA fragments.

Figure S2. The plasmid maps for **a.** empty Yip 128, **b.** empty Yip 211 for the overexpression of **c**. *ALD5PAD1*, and **d**. Plasmid for the overexpression of *ATF1* and *ATF2*.

Figure S3: Proposed conversion route of ferulic acid into other phenolic compounds. **ii:** Conversion product profile of ferulic acid for **a.** *APT_1*, **b.** *B_CALD*, **c.** *SC_ald5Δ*, and **d.** the control strain

Figure S4: Proposed conversion route of p-coumaric acid into other phenolic compounds. **ii:** Conversion product profile of p-coumaric acid for **a.** *APT_1*, **b.** *B_CALD*, **c.** *SC_ald5Δ*, and **d.** the control strain.

Figure S5. Plots for conversion products of coniferyl aldehyde ferulic acid and p-coumaric acid. **i.***APT_1* CA products, **ii.** *B_CALD* CA products, **iii.** *SC_ald5Δ* CA products, **iv.** CTRL CA products, **v.** *APT_1* FA products, **vi.** *B_CALD* FA products, **vii.** *SC_ald5Δ* FA and products, **viii.** CTRL FA products, **ix.** *APT_1* pCA products, **x.** *B_CALD* pCA products, **xi.** *SC_ald5Δ* pCA products, **xii.** CTRL FA products.

i

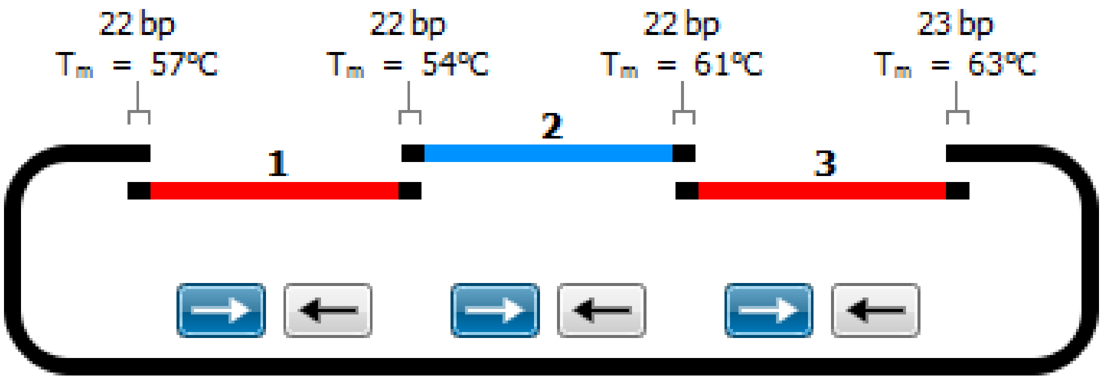

ii

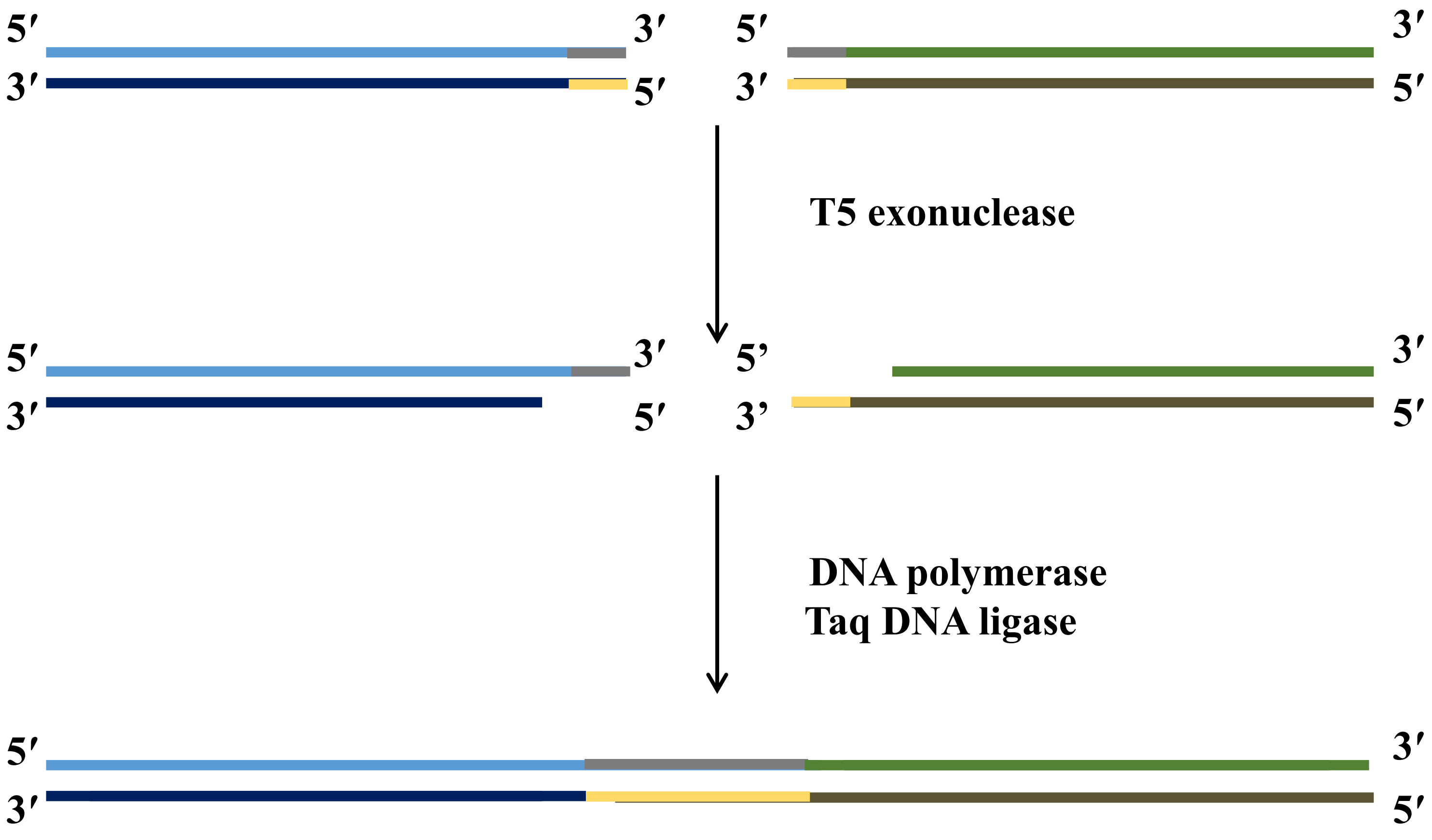

Figure **S1**. **i** Outline of plasmid assembled via Gibson protocol. **ii**. The principle of Gibson assembly for assembling DNA fragments.

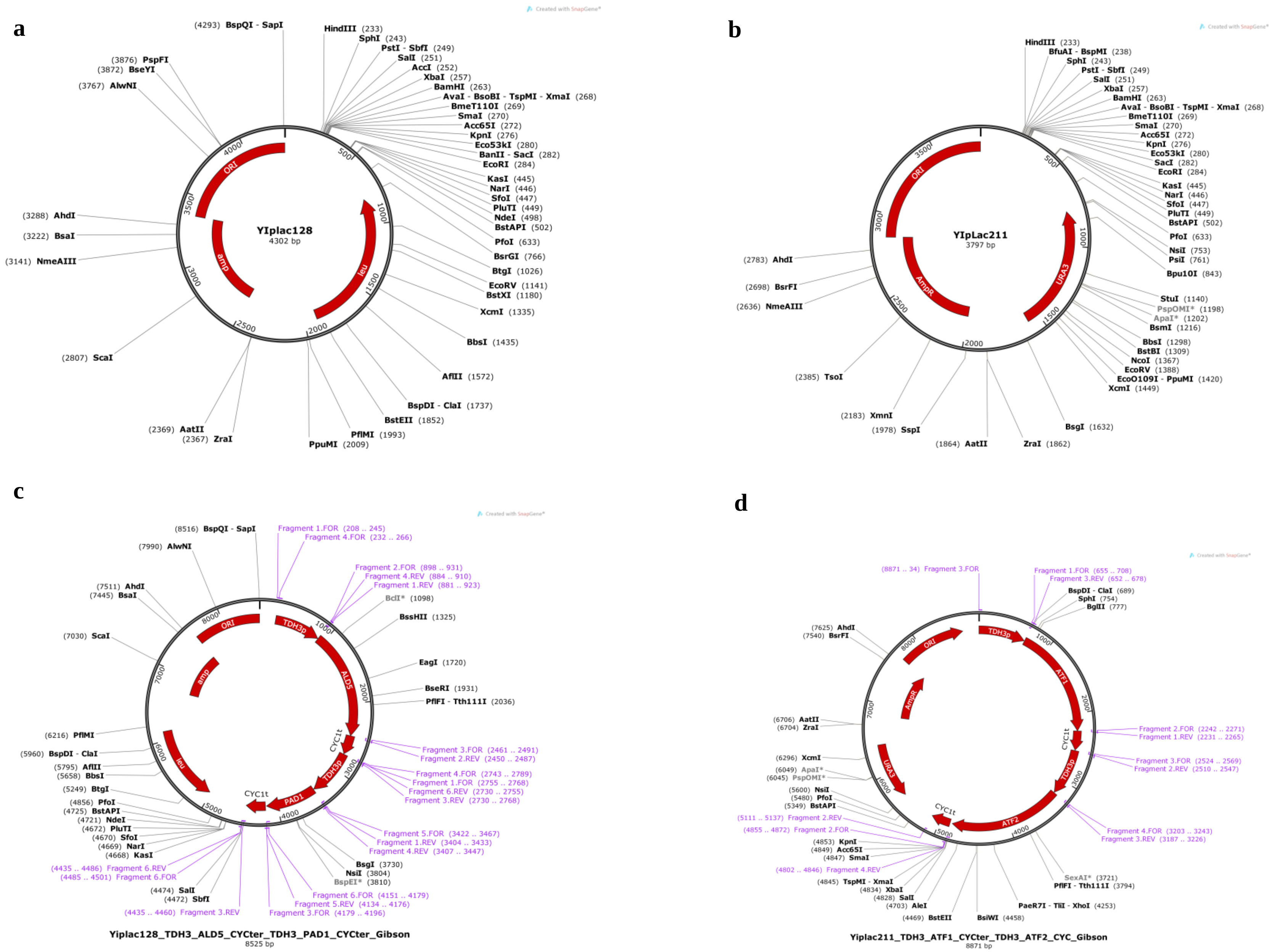

Figure S2. The plasmid maps for **a.** empty Yip 128, **b.** empty Yip 211 for the overexpression of **c**. *ALD5PAD1*, and **d**. Plasmid for the overexpression of *ATF1* and *ATF2*.

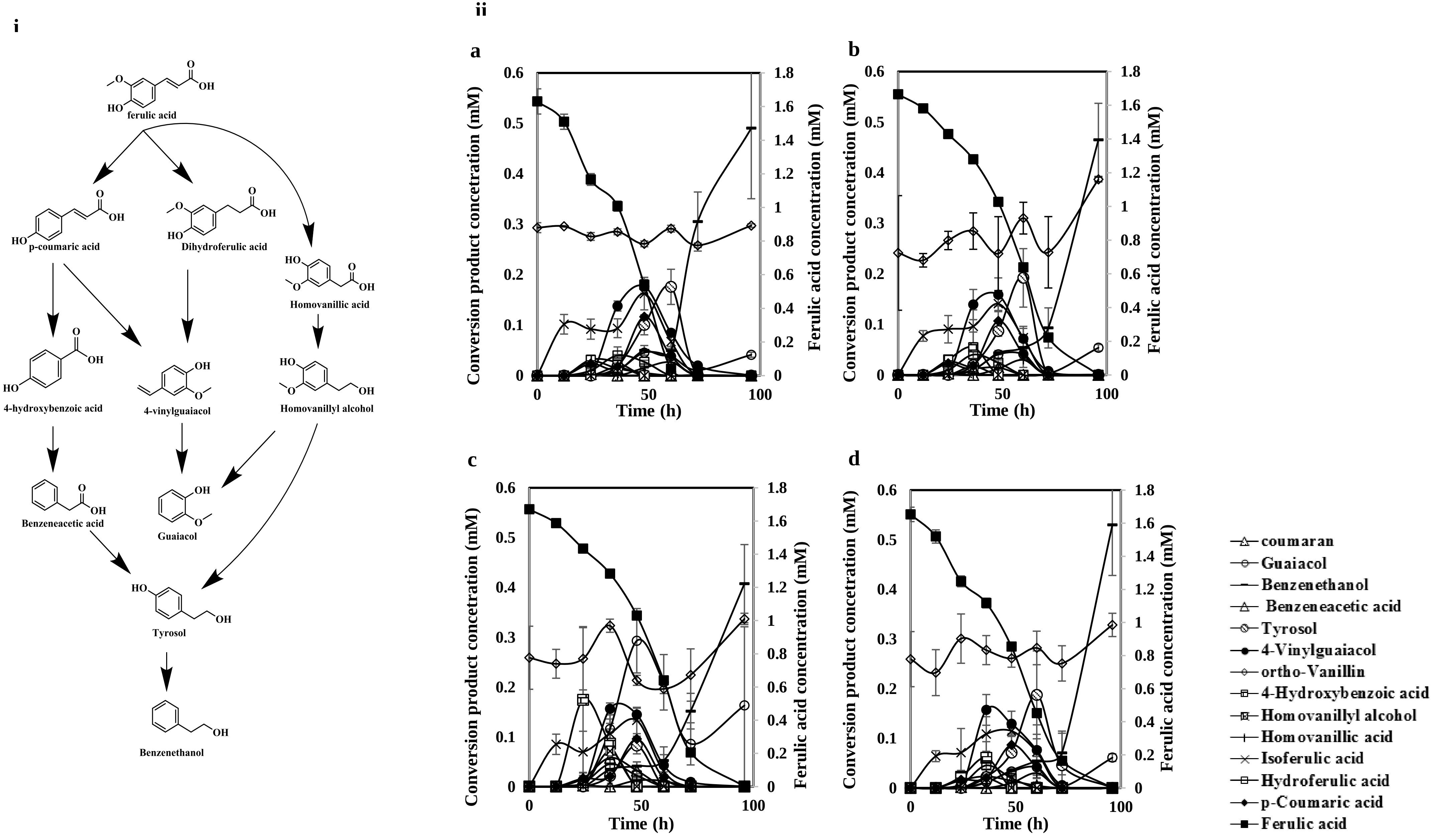

i
ferulic acid
p-coumaric acid
Dihydroferulic acid
Homovanillic acid
4-hydroxybenzoic acid
4-vinylguaiacol
Homovanillyl alcohol
Benzeneacetic acid
Guaiacol
Tyrosol
Benzenethanol
ii
a
b
c
d
Conversion product concetration (mM)
Ferulic acid concentration (mM)
Time (h)
coumaran
Guaiacol
Benzenethanol
Benzeneacetic acid
Tyrosol
4-Vinylguaiacol
ortho-Vanillin
4-Hydroxybenzoic acid
Homovanillyl alcohol
Homovanillic acid
Isoferulic acid
Hydroferulic acid
p-Coumaric acid
Ferulic acid

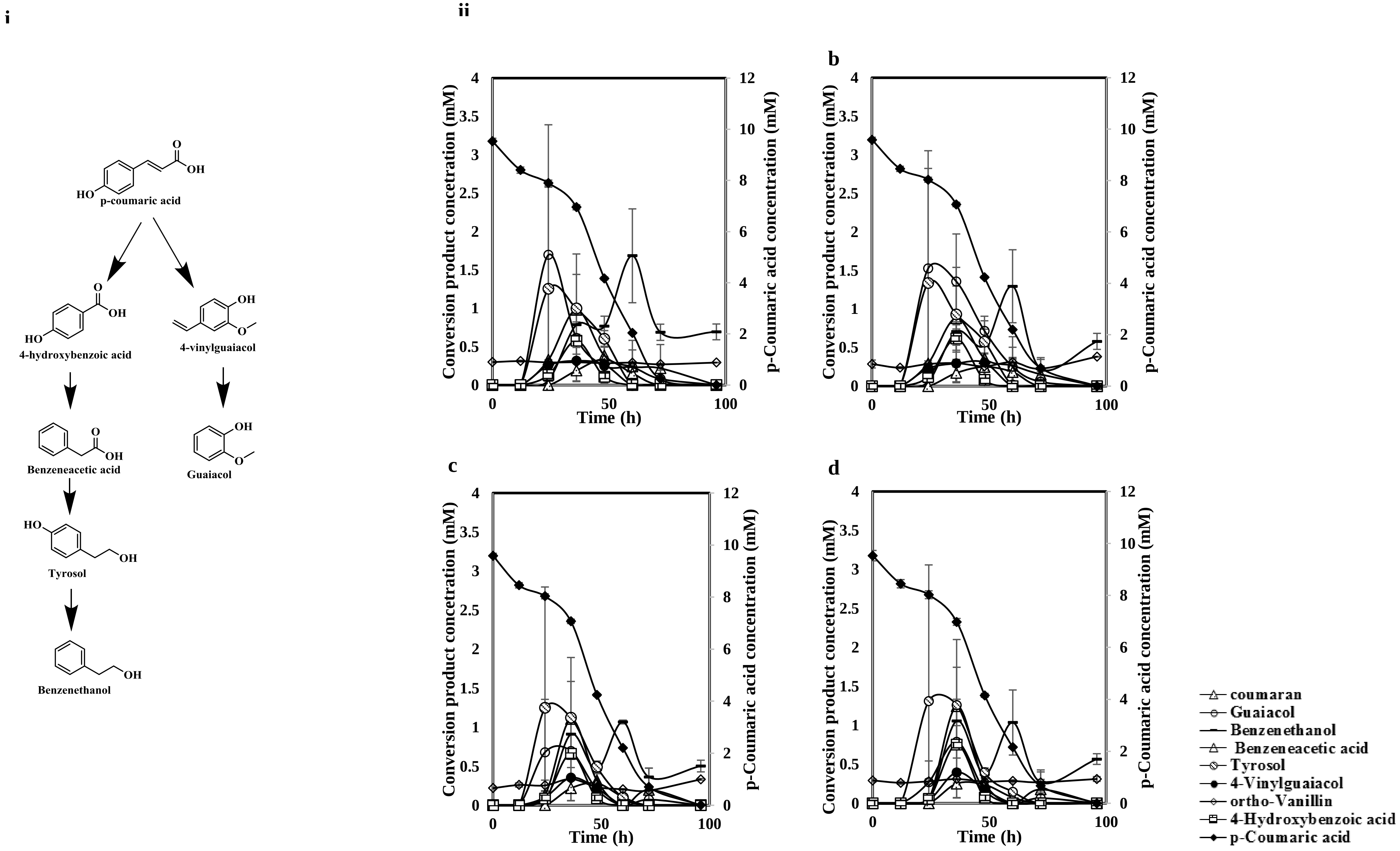
i
ii
p-coumaric acid
4-hydroxybenzoic acid
4-vinylguaiacol
Benzeneacetic acid
Guaiacol
Tyrosol
Benzenethanol
b
c
d
Conversion product concetration (mM)
p-Coumaric acid concentration (mM)
Time (h)
coumaran
Guaiacol
Benzenethanol
Benzeneacetic acid
Tyrosol
4-Vinylguaiacol
ortho-Vanillin
4-Hydroxybenzoic acid
p-Coumaric acid

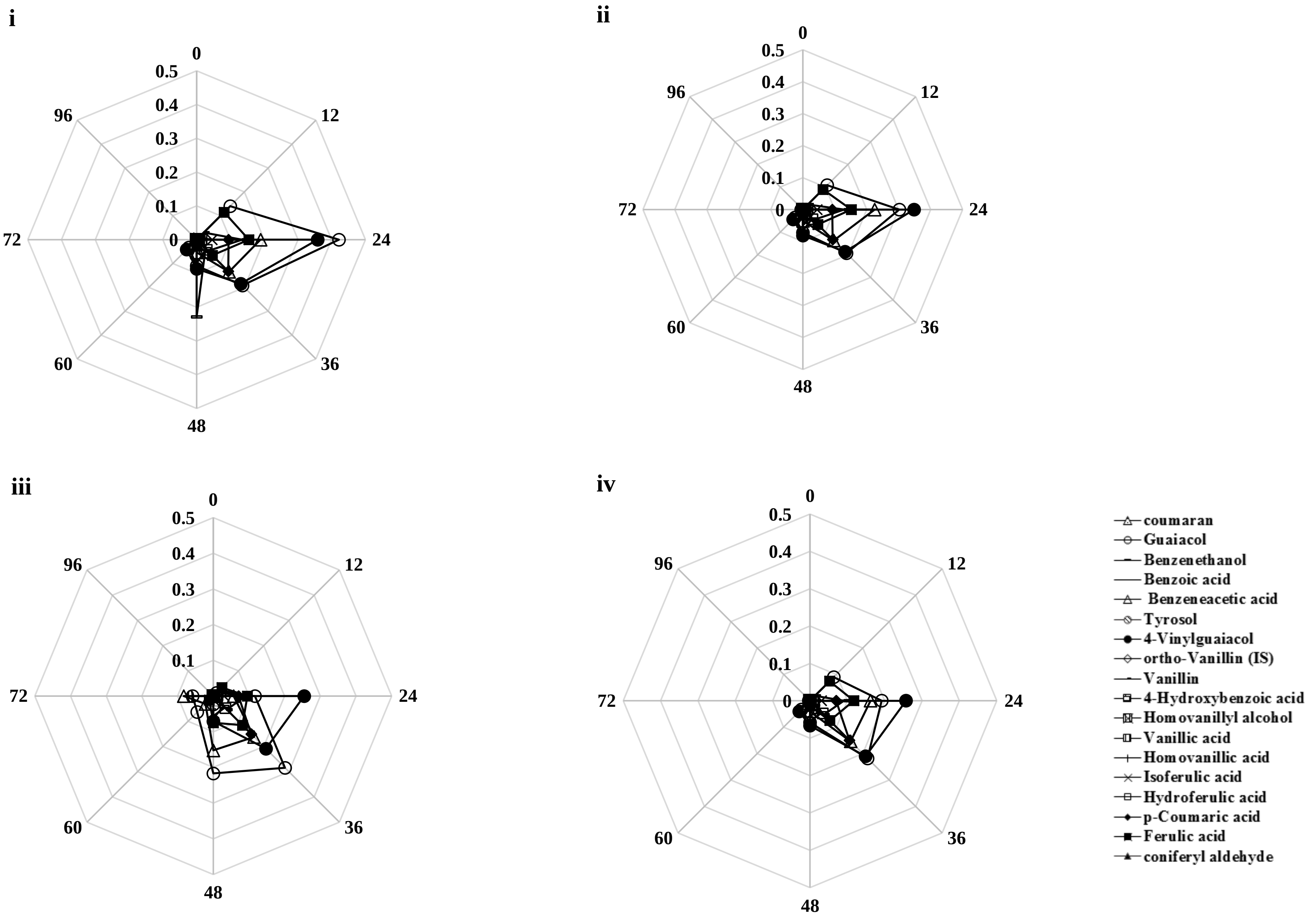
i
ii
iii
iv
0
12
24
36
48
60
72
96
0.5
0.4
0.3
0.2
0.1
coumaran
Guaiacol
Benzenethanol
Benzoic acid
Benzeneacetic acid
Tyrosol
4-Vinylguaiacol
ortho-Vanillin (IS)
Vanillin
4-Hydroxybenzoic acid
Homovanillyl alcohol
Vanillic acid
Homovanillic acid
Isoferulic acid
Hydroferulic acid
p-Coumaric acid
Ferulic acid
coniferyl aldehyde

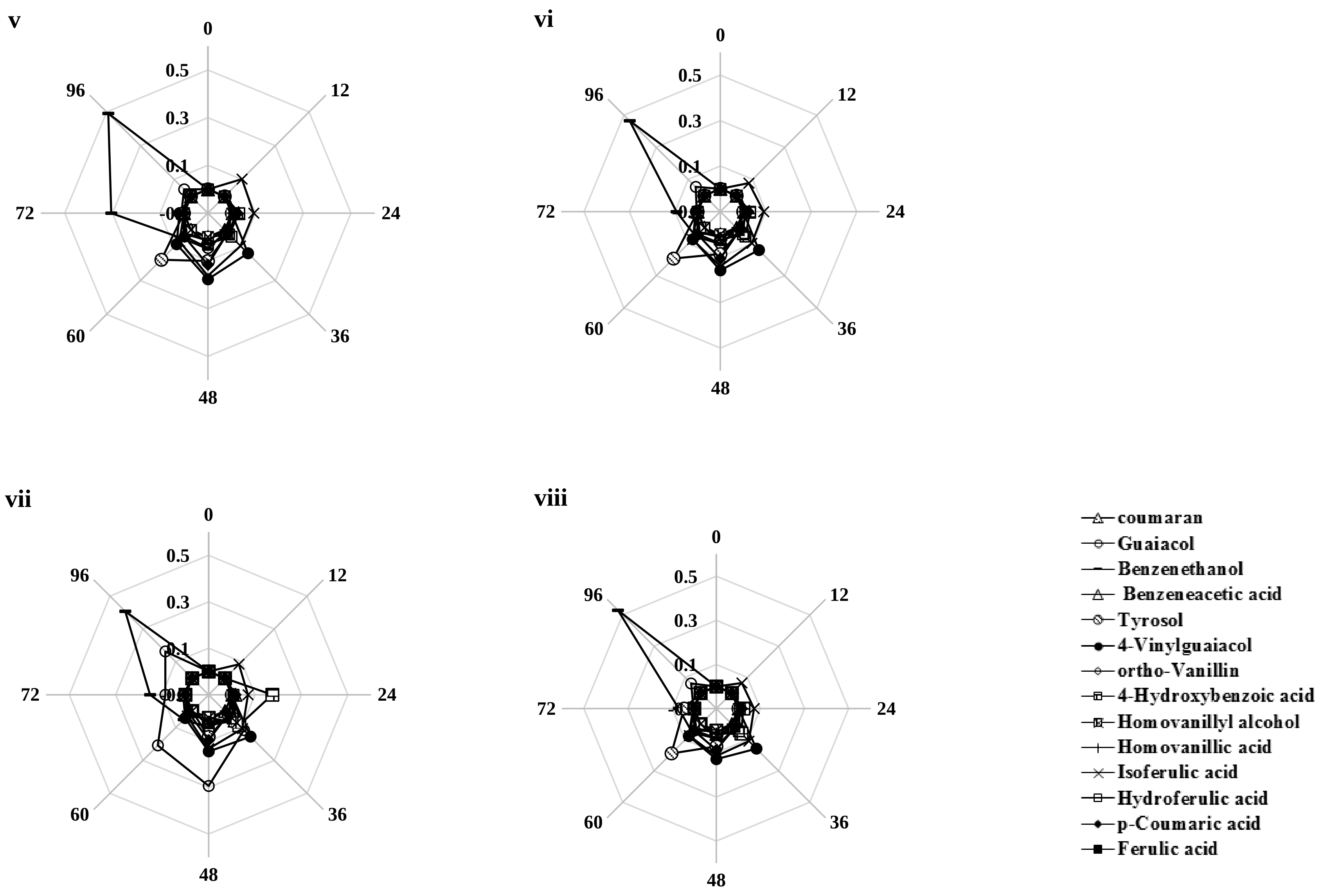
v
vi
vii
viii
0
12
24
36
48
60
72
96
0.5
0.3
0.1
coumaran
Guaiacol
Benzenethanol
Benzeneacetic acid
Tyrosol
4-Vinylguaiacol
ortho-Vanillin
4-Hydroxybenzoic acid
Homovanillyl alcohol
Homovanillic acid
Isoferulic acid
Hydroferulic acid
p-Coumaric acid
Ferulic acid

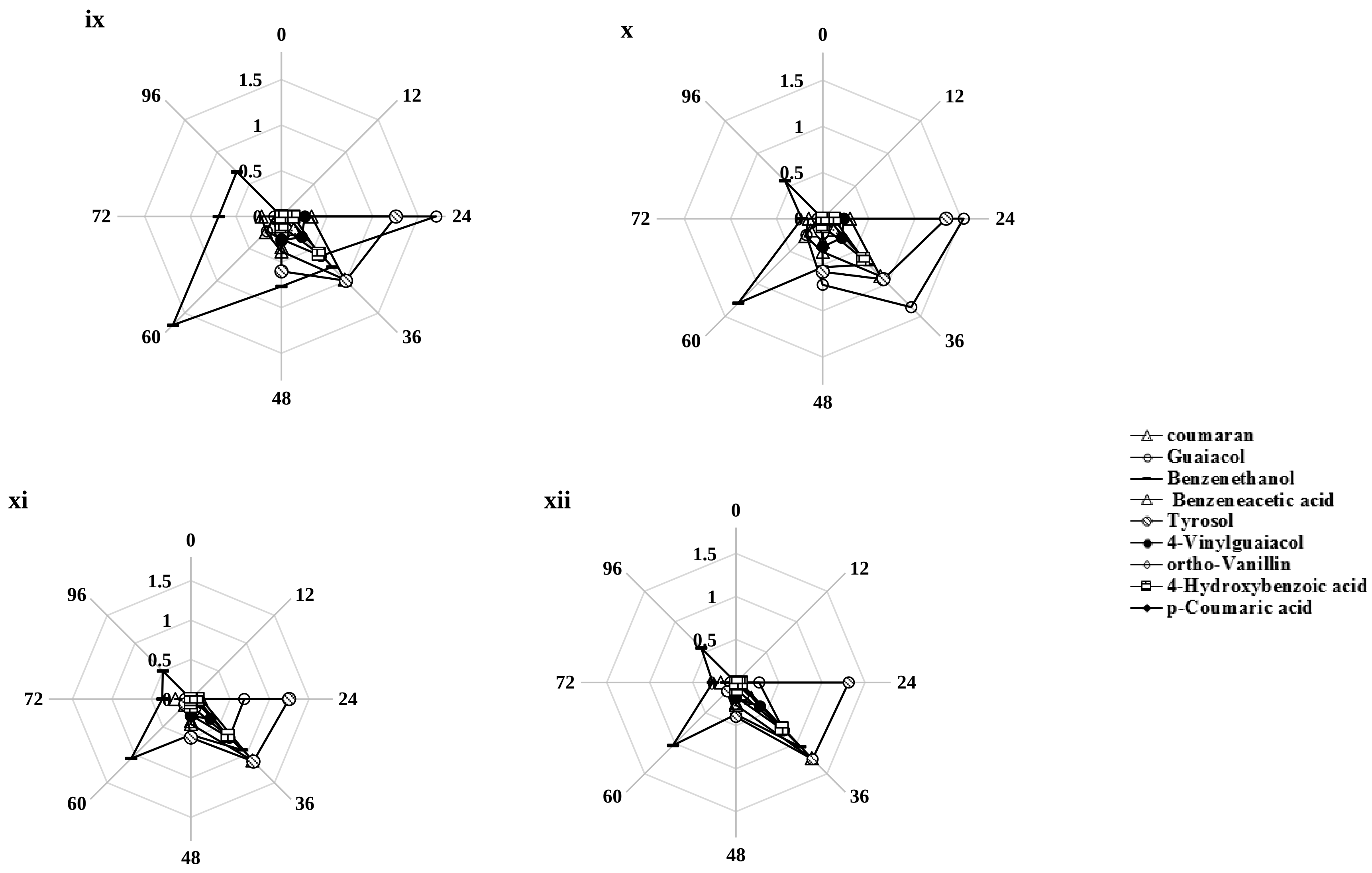

Figure S3. Plots for conversion products of coniferyl aldehyde ferulic acid and p-coumaric acid. **i.***APT_1* CA products, **ii.** *B_CALD* CA products, **iii.** *SC_ald5Δ* CA products, **iv.** CTRL CA products, **v.** *APT_1* FA products, **vi.** *B_CALD* FA products, **vii.** *SC_ald5Δ* FA and products, **viii.** CTRL FA products, **ix.** *APT_1* pCA products, **x.** *B_CALD* pCA products, **xi.** *SC_ald5Δ* pCA products, **xii.** CTRL FA products.

Table S1. Conversion products of coniferyl aldehyde by *APT_1*, *B_CALD*, *SC_ald5Δ* and control strains.

APT_1																		
**Time (h)**	**coumaran**	**Guaiacol**	**Benzenethanol**	**Benzoic acid**	**Benzeneacetic acid**	**Tyrosol**	**4-Vinylguaiacol**	**ortho-Vanillin (IS)**	**Vanillin**	**4-Hydroxybenzoic acid**	**Homovanillyl alcohol**	**Vanillic acid**	**Homovanillic acid**	**Isoferulic acid**	**coniferyl aldehyde**	**Hydroferulic acid**	**p-Coumaric acid**	**Ferulic acid**
**0**	0	0	0	0	0	0	0	0.311 ± 0.013	0	0	0	0	0	0	0.90.02	0	0	0
**12**	0	0.14 ± 0.012	0	0	0	✓	0	0.321 ± 0.01	0.03 ± 0.01	0	0	✓	0	0.008 ± 0.002	0.4 ± 0.17	0	0	0.115 ± 0.03
**24**	✓	0.421 ± 0.04	✓	✓	0.189 ± 0.027	0.01 ±0.000	0.358 ± 0.015	0.312 ± 0.01	0.135 ± 0.013	✓	0	✓	✓	0.046 ± 0.02	0.065 ± 0.04	0.015 ± 0.004	0.095 ± 0.01	0.155 ± 0.03
**36**	✓	0.192 ± 0.011	0.037 ± 0.008	0.04 ± 0.007	0.135 ± 0.02	0.015 ± 0.004	0.184 ± 0.012	0.306 ± 0.01	0.048 ± 0.013	0.007 ± 0.001	✓	✓	0.006 ±	0	0	0.042 ± 0.005	0.132 ±0.003	0.066 ± 0.01
**48**	0	0.078 ± 0.007	0.228 ± 0.037	0.07 ± 0.001	0.038 ± 0.002	0	0.087 ± 0.01	0.3 ± 0.004	✓	✓	0.013 ± 0.01	0	0.009 ±	0	0	0	0	0.019 ± 0.01
**60**	0	0.033 ±0.004	✓	✓	0.016 ± 0.002	0	0.042 ± 0.003	0.293 ± 0.005	0	0	✓	0	0	0	0	0	0	0
**72**	0	0	0	✓	0	0	✓	0.286 ± 0.01	0	0	✓	0	0	0	0	0	0	0
**96**	0	0	0	0	0	0	0	0.289 ± 0.01	0	0	0	0	0	0	0	0	0	0

"✓" implies the presence of a compound in quantities that cannot be reliably quantified
"0" implies the complete absence of a compound

B_CALD

Time (h)	coumaran	Guaiacol	Benzenethanol	Benzoic acid	Benzeneacetic acid	Tyrosol	4-Vinylguaiacol	ortho-Vanillin	Vanillin	4-Hydroxybenzoic acid	Homovanillyl alcohol	Vanillic acid	Homovanillic acid	Isoferulic acid	coniferyl aldehyde	Hydroferulic acid	p-Coumaric acid	Ferulic acid
**0**	0	0	0	0	0	0	0	0.269 ± 0.03	0	0	0	0	0	0	0.924 ± 0.01	0	0	0
**12**	0	0.108 ± 0.015	0	0	0	✓	0	0.246 ± 0.02	0.022 ± 0.03	0	0	✓	0	0.006 ± 0.002	0.637 ± 0.01	0	0	0.089 ± 0.03
**24**	✓	0.302 ± 0.05	✓	✓	0.225 ± 0.02	0.009 ± 0.000	0.348 ±0.05	0.279 ± 0.01	0.129 ± 0.01	✓	0	✓	✓	0.044 ± 0.02	0.116 ± 0.001	0.014 ± 0.004	0.092 ± 0.02	0.152 ± 0.04
**36**	✓	0.193 ± 0.04	0.04 ± 0.014	0.036 ±0.01	0.137 ± 0.04	0.015 ± 0.002	0.186 ± 0.04	0.283 ± 0.01	0.046 ± 0.01	0.007 ± 0.0002	✓	✓	0.005 ± 0.0001	0	0	0.042 ± 0.01	0.132 ± 0.02	0.066 ± 0.01
**48**	0	0.072 ± 0.02	0.067 ± 0.01	0.049 ±0.000	0.035 ± 0.01	0	0.081 ± 0.03	0.233 ± 0.02	✓	✓	0.011 ± 0.004	0	0.006 ± 0.0001	0	0	0	0	0.017 ± 0.005
**60**	0	0.035 ±0.004	0.014 ± 0.000	✓	0.017 ± 0.002	0	0.043 ± 0.003	0.29 ± 0.02	0	0	✓	0	0	0	0	0	0	0
**72**	0	0.005 ± 0.001	✓	✓	0	0	✓	0.233 ± 0.04	0	0	✓	0	0	0	0	0	0	0
**96**	0	0	0	0	0	0	0	0.288 ± 0.04	0	0	0	0	0	0	0	0	0	0

"✓" implies the presence of a compound in quantities that cannot be reliably quantified
"0" implies the complete absence of a compound

SC_ald5Δ

Time (h)	coumaran	Guaiacol	Benzenethanol	Benzoic acid	Benzeneacetic acid	Tyrosol	4-Vinylguaiacol	ortho-Vanillin	Vanillin	4-Hydroxybenzoic acid	Homovanillyl alcohol	Vanillic acid	Homovanillic acid	Isoferulic acid	coniferyl aldehyde	Hydroferulic acid	p-Coumaric acid	Ferulic acid
**0**	0	0	0	0	0	0	0	0.254 ± .03	0	0	0	0	0	0	0.9 ± 0.01	0	0	0
**12**	0	0.013 ± 0.01	0	0	0	✓	0	0.268 ± .03	0.025 ± 0.01	0	0	✓	0	0.006 ± 0.003	0.621 ± 0.01	0	0	0.029 ± 0.01
**24**	✓	0.117 ± 0.02	✓	✓	0.057 ± 0.02	0.004 ± 0.002	0.255 ± 0.12	0.282 ± 0.02	0.091 ± 0.03	✓	0	✓	✓	0.03 ± 0.01	0.213 ± 0.01	0.011 ± 0.005	0.071 ± 0.03	0.09 ± 0.03
**36**	✓	0.284 ± 0.09	0.041 ± 0.01	0.046 ± 0.01	0.162 ± 0.06	0.013 ± 0.003	0.209 ± 0.01	0.302 ± 0.04	0.054 ± 0.014	0.007 ± 0.001	✓	✓	0.007 ± 0.001	0	0	0.048 ± 0.01	0.15 ± 0.01	0.11 ± 0.01
**48**	0	0.217 ±0.01	0.062 ± 0.01	0.056 ± 0.000	0.152 ± 0.02	0.024 ±	0.071 ± 0.01	0.245 ± 0.005	✓	✓	0.01 ± 0.005	0	0.007 ± 0.001	0	0	0	0	0.07 ± 0.01
**60**	✓	0.064 ± 0.01	✓	✓	0.031 ± 0.001	0	✓	0.257 ± 0.03	✓	✓	✓	✓	✓	✓	0	✓	✓	0.011 ± 0.01
**72**	0	0.058 ± 0.05	0	✓	0.083 ± 0.01	✓	✓	0.287 ± 0.02	0	0	✓	0	0	0	0	0	0	0
**96**	0	0	0	0	0	0	0	0.285 ± 0.04	0	0	0	0	0	0	0	0	0	0

"✓" implies the presence of a compound in quantities that cannot be reliably quantified
"0" implies the complete absence of a compound

	Ctrl																	
**Time (h)**	**coumaran**	**Guaiacol**	**Benzenethanol**	**Benzoic acid**	**Benzeneacetic acid**	**Tyrosol**	**4-Vinylguaiacol**	**ortho-Vanillin**	**Vanillin**	**4-Hydroxybenzoic acid**	**Homovanillyl alcohol**	**Vanillic acid**	**Homovanillic acid**	**Isoferulic acid**	**coniferyl aldehyde**	**Hydroferulic acid**	**p-Coumaric acid**	**Ferulic acid**
**0**	0	0	0	0	0	0	0	0.316 ± 0.04	0	0	0	0	0	0	0.884 ± 0.01	0	0	0
**12**	0	0.09 ± 0.02	0	0	0	✓	0	0.248 ± 0. 05	0.019 ± 0.004	0	0	✓	0	0.005 ± 0.002	0.498 ± 0.01	0	0	0.074 ± 0.03
**24**	✓	0.192 ± 0.08	✓	✓	0.162 ± 0.08	0.004 ± 0.002	0.257 ± 0.14	0.261 ± 0.08	0.091 ± 0.04	✓	0	✓	✓	0.03 ± 0.02	0.131 ± 0.001	0.01 ± 0.005	0.07 ± 0.04	0.117 ± 0.08
**36**	✓	0.218 ± 0.04	0.045 ± 0.01	0.041 ± 0.01	0.154 ± 0.04	0.017 ± 0.000	0.21 ± 0.04	0.323 ± 0.01	0.052 ± 0.008	0.008 ± 0.003	✓	✓	0.006 ± 0.0002	0	0	0.048 ± 0.008	0.149 ± 0.02	0.075 ± 0.01
**48**	0	0.058 ± 0.02	0.054 ± 0.01	0.039 ± 0.000	0.028 ± 0.01	0	0.066 ± 0.02	0.251 ± 0.05	✓	✓	0.009 ± 0.003	0	0.005 ± 0.000	0	0	0	0	0.014 ± 0.004
**60**	0	0.032 ± 0.01	✓	✓	0.016 ± 0.004	0	0.041 ± 0.01	0.281 ± 0.02	0	0	✓	0	0	0	0	0	0	0
**72**	0	0.004 ± 0.003	✓	✓	0	0	✓	0.269 ± 0.07	0	0	✓	0	0	0	0	0	0	0
**96**	0	0	0	0	0	0	0	0.295 ± 0.04	0.002 ± 0.	0	0	0	0	0	0	0	0	0

"✓" implies the presence of a compound in quantities that cannot be reliably quantified
"0" implies the complete absence of a compound

Table S2. Conversion products of ferulic acid by *APT_1*, *B_CALD*, *SC_ald5Δ* and control strains.

APT_1

**Time (h)**	**coumaran**	**Guaiacol**	**Benzeneethanol**	**Benzeneacetic acid**	**Tyrosol**	**4-Vinylguaiacol**	**ortho-Vanillin**	**4-Hydroxybenzoic acid**	**Homovanillyl alcohol**	**Homovanillic acid**	**Isoferulic acid**	**Hydroferulic acid**	**p-Coumaric acid**	**Ferulic acid**
**0**	0	0	0	0	0	0	0.293 ± 0.01	0	0	0	0	0	0	1.631 ± 0.08
**12**	0	✓	✓	✓	0	✓	0.296 ± 0.002	0	0	0	0.102 ± 0.02	✓	✓	1.510 ± 0.05
**24**	0.003 ± 0.003	✓	✓	0.028 ± 0.01	✓	✓	0.276 ± 0.01	0.032 ± 0.003	✓	0.002 ± 0.003	0.092 ± 0.02	0.010 ± 0.003	0.024 ± 0.004	1.167 ± 0.04
**36**	✓	0.010 ± 0.002	0.009 ± 0.003	0.014 ± 0.002	0.019 ± 0.005	0.138 ± 0.01	0.285 ± 0.01	0.026 ± 0.003	0.016 ± 0.001	✓	0.094 ± 0.02	0.040 ± 0.002	0.018 ± 0.002	1.008 ± 0.03
**48**	✓	0.046 ± 0.006	0.050 ± 0.009	0.006 ± 0.005	0.100 ± 0.02	0.177 ± 0.01	0.261 ± 0.01	✓	✓	0.015 ± 0.007	0.163 ± 0.03	0.027 ± 0.003	0.117 ± 0.004	0.540 ± 0.02
**60**	✓	0.038 ± 0.005	0.050 ± 0.008	0	0.176 ± 0.04	0.085 ± 0.007	0.291 ± 0.01	0	0	0.027 ± 0.01	0.068 ± 0.01	0	0.039 ± 0.003	0.041 ± 0.001
**72**	0.001 ± 0.001	0.011 ± 0.005	0.305 ± 0.06	0.001 ± 0.001	0.001 ± 0.001	0.020 ± 0.004	0.258 ± 0.004	0.001 ± 0.001	0.001 ± 0.001	✓	0.001 ± 0.001	0.001 ± 0.001	0.001 ± 0.001	0
**96**		0.041 ± 0.005	0.491 ± 0.1	0	0	0	0.297 ± 0.004	0	0	0	0	0	0	0

"✓" implies the presence of a compound in quantities that cannot be reliably quantified
"0" implies the complete absence of a compound

B_CALD

**Time (h)**	**coumaran**	**Guaiacol**	**Benzeneethanol**	**Benzeneacetic acid**	**Tyrosol**	**4-Vinylguaiacol**	**ortho-Vanillin**	**4-Hydroxybenzoic acid**	**Homovanillyl alcohol**	**Homovanillic acid**	**Isoferulic acid**	**Hydroferulic acid**	**p-Coumaric acid**	**Ferulic acid**
**0**	0	0	0	0	0	0	0.241 ± 0.1	0	0	0	0	0	0	1.665 ± 0.0
**12**	0	✓	✓	✓	0	✓	0.227 ± 0.001	0	0	0	0.077 ± 0.01	✓	✓	1.581 ± 0.02
**24**	0.003 ± 0.004	✓	✓	0.026 ± 0.01	✓	✓	0.266 ± 0.02	0.031 ± 0.005	✓	0.002 ± 0.003	0.090 ± 0.03	0.010 ± 0.003	0.023 ± 0.003	1.429 ± 0.02
**36**	✓	0.011 ± 0.004	0.010 ± 0.004	0.014 ± 0.003	0.019 ± 0.008	0.139 ± 0.03	0.285 ± 0.04	0.055 ± 0.03	0.017 ± 0.003	✓	0.096 ± 0.03	0.040 ± 0.007	0.018 ± 0.003	1.279 ± 0.01
**48**	✓	0.041 ± 0.008	0.043 ± 0.003	0.015 ± 0.01	0.087 ± 0.007	0.159 ± 0.03	0.240 ± 0.07	✓	✓	0.012 ± 0.003	0.141 ± 0.02	0.024 ± 0.005	0.106 ± 0.03	1.026 ± 0.01
**60**	✓	0.041 ± 0.009	0.054 ± 0.014	0	0.192 ± 0.006	0.072 ± 0.02	0.310 ± 0.03	0	0	0.030 ± 0.02	0.074 ± 0.02	0	0.042 ± 0.006	0.638 ± 0.006
**72**	0	0.004 ± 0.001	0.093 ± 0.004	0.001 ± 0.001	0.001 ± 0.001	0.008 ± 0.002	0.243 ± 0.07	0.001 ± 0.001	0	✓	0	0	0	0.222 ± 0.02
**96**	0	0.054 ± 0.006	0.465 ± 0.007	0	0	0	0.387 ± 0.005	0	0	0	0	0	0	0

“✓” implies the presence of a compound in quantities that cannot be reliably quantified
“0” implies the complete absence of a compound

SC_ald5Δ

Time (h)	coumaran	Guaiacol	Benzeneethanol	Benzeneacetic acid	Tyrosol	4-Vinylguaiacol	ortho-Vanillin	4-Hydroxybenzoic acid	Homovanillyl alcohol	Homovanillic acid	Isoferulic acid	Hydroferulic acid	p-Coumaric acid	Ferulic acid
**0**	0	0	0	0	0	0	0.259 ± 0.06	0	0	0	0	0	0	1.670 ± 0.02
**12**	0	✓	✓	✓	0	✓	0.247 ± 0.03	0	0	0	0.085 ± 0.02	✓	✓	1.587 ± 0.02
**24**	0.004 ± 0.003	✓	✓	0.016 ± 0.004	✓	✓	0.257 ± 0.06	0.174 ± 0.15	✓	0.003 ± 0.005	0.070 ± 0.04	0.007 ± 0.003	0.016 ± 0.006	1.434 ± 0.02
**36**	✓	0.115 ± 0.02	0.034 ± 0.03	0.058 ± 0.05	0.021 ± 0.005	0.156 ± 0.01	0.323 ± 0.01	0.086 ± 0.06	0.071 ± 0.07	✓	0.106 ± 0.02	0.045 ± 0.001	0.020 ± 0.001	1.283 ± 0.02
**48**	✓	0.293 ± 0.070	0.041 ± 0.008	0.032 ± 0.02	0.082 ± 0.02	0.145 ± 0.01	0.213 ± 0.01	✓	✓	0.012 ± 0.006	0.133 ± 0.03	0.022 ± 0.003	0.095 ± 0.006	1.030 ± 0.01
**60**	✓	0.209 ± 0.060	0.052 ± 0.003	0.001 ± 0.001	✓	0.043 ± 0.02	0.196 ± 0.01	0	0	0.011 ± 0.01	0.031 ± 0.02	0	0.017 ± 0.012	0.640 ± 0.01
**72**	0.001 ± 0.001	0.086 ± 0.04	0.152 ± 0.04	0	0	0.009 ± 0.007	0.224 ± 0.05	0	0	✓	0	0	0	0.206 ± 0.003
**96**	0	0.163 ± 0.020	0.408 ± 0.08	0	0	0	0.337 ± 0.01	0	0	0	0	0	0	0

"✓" implies the presence of a compound in quantities that cannot be reliably quantified
"0" implies the complete absence of a compound

CTRL

Time (h)	coumaran	Guaiacol	Benzeneethanol	Benzeneacetic acid	Tyrosol	4-Vinylguaiacol	ortho-Vanillin	4-Hydroxybenzoic acid	Homovanillyl alcohol	Homovanillic acid	Isoferulic acid	Hydroferulic acid	p-Coumaric acid	Ferulic acid
**0**	0	0	0	0	0	0	0.259 ± 0.06	0	0	0	0	0	0	1.653 ± 0.04
**12**	0	✓	✓	✓	0	✓	0.232 ± 0.05	0	0	0	0.064 ± 0.01	✓	✓	1.520 ± 0.04
**24**	0.002 ± 0.002	✓	✓	0.015 ± 0.002	✓	✓	0.301 ± 0.05	0.022 ± 0.01	✓	0.001 ± 0.002	0.071 ± 0.05	0.007 ± 0.003	0.016 ± 0.007	1.249 ± 0.03
**36**	✓	0.012 ± 0.004	0.011 ± 0.004	0.023 ± 0.008	0.022 ± 0.008	0.157 ± 0.03	0.278 ± 0.03	0.061 ± 0.03	0.019 ± 0.003	✓	0.109 ± 0.04	0.045 ± 0.007	0.020 ± 0.003	1.118 ± 0.03
**48**	✓	0.033 ± 0.006	0.036 ± 0.003	0.014 ± 0.008	0.071 ± 0.004	0.129 ± 0.03	0.261 ± 0.02	✓	✓	0.010 ± 0.003	0.115 ± 0.01	0.020 ± 0.004	0.087 ± 0.02	0.854 ± 0.02
**60**	✓	0.042 ± 0.02	0.054 ± 0.03	0.005 ± 0.007	0.188 ± 0.09	0.076 ± 0.05	0.282 ± 0.03	0	0	0.030 ± 0.02	0.073 ± 0.04	0	0.042 ± 0.02	0.453 ± 0.01
**72**	0	0.003 ± 0.000	0.070 ± 0.04	0	0.05 ± 0.05	0.006 ± 0.002	0.251 ± 0.04	0	0	✓	0	0	0	0.164 ± 0.004
**96**	0	0.061 ± 0.008	0.530 ± 0.1	0	0	0	0.328 ± 0.02	0	0	0	0	0	0	0

"✓" implies the presence of a compound in quantities that cannot be reliably quantified
"0" implies the complete absence of a compound

Table S3. Conversion products of *p*-coumaric acid by *APT_1*, *B_CALD*, *SC_ald5Δ* and control strains.

APT_1

Time (h)	coumaran	Guaiacol	Benzenethanol	Benzeneacetic acid	Tyrosol	4-Vinylguaiacol	ortho-Vanillin	4-Hydroxybenzoic acid	p-Coumaric acid
**0**	0	0	0	0	0	0	0.299 ± 0.01	0	9.529 ± 0.12
**12**	0	0	0	0	0	0	0.312 ± 0.01	0	8.404 ± 0.12
**24**	✓	1.696 ± 0.23	0.062 ± 0.06	0.329 ± 0.05	1.254 ± 0.13	0.255 ± 0.00	0.295 ± 0.02	0.131 ± 0.10	7.899 ± 0.11
**36**	0.189 ± 0.01	0.613 ± 0.21	0.786 ± 0.14	0.979 ± 0.12	0.997 ± 0.07	0.316 ± 0.2	0.297 ± 0.01	0.576 ± 0.09	6.951 ± 0.10
**48**	0.335 ± 0.02	0.249 ± 0.01	0.766 ± 0.13	0.385 ± 0.03	0.602 ± 0.11	0.265 ± 0.2	0.285 ± 0.02	0.108 ± 0.07	4.169 ± 0.06
**60**	0.153 ± 0.01	0.229 ± 0.13	1.685 ± 0.61	0.241 ± 0.03	0	0	0.292 ± 0.001	0	2.040 ± 0.03
**72**	0	0.081 ± 0.01	0.688 ± 0.11	0.219 ± 0.03	0.011 ± 0.01	0	0.271 ± 0.01	0.007 ± 0.01	0.235 ± 0.01
**96**	0	0	0.693 ± 0.1	0	0	0	0.293 ± 0.03	0	0

"✓" implies the presence of a compound in quantities that cannot be reliably quantified
"0" implies the complete absence of a compound

B_CALD									
**Time (h)**	**coumaran**	**Guaiacol**	**Benzenethanol**	**Benzeneacetic acid**	**Tyrosol**	**4-Vinylguaiacol**	**ortho-Vanillin**	**4-Hydroxybenzoic acid**	**p-Coumaric acid**
**0**	0	0	0	0	0	0	0.287 ± 0.05	0	9.587 ± 0.10
**12**	0	0	0	0	0	0	0.239 ± 0.01	0	8.454 ± 0.010
**24**	✓	1.526 ± 0.10	0.056 ± 0.06	0.296 ± 0.04	1.337 ± 0.14	0.230 ± 0.00	0.287 ± 0.04	0.120 ± 0.01	8.032 ± 0.10
**36**	0.170 ± 0.12	1.357 ± 0.62	0.707 ± 0.12	0.881 ± 0.02	0.930 ± 0.06	0.294 ± 0.02	0.298 ± 0.05	0.618 ± 0.02	7.067 ± 0.10
**48**	0.249 ± 0.17	0.717 ± 0.19	0.525 ± 0.20	0.361 ± 0.04	0.577 ± 0.03	0.304 ± 0.03	0.257 ± 0.06	0.081 ± 0.05	4.238 ± 0.05
**60**	0.183 ± 0.18	0.252 ± 0.03	1.295 ± 0.50	0.265 ± 0.02	0.008 ± 0.008	0	0.311 ± 0.04	0	2.202 ± 0.02
**72**	0	0.056 ± 0.008	0.227 ± 0.01	0.153 ± 0.0	0	0	0.226 ± 0.04	0	0.701 ± 0.01
**96**	0	0	0.580 ± 0.01	0	0	0	0.381 ± 0.004	0	0

"✓" implies the presence of a compound in quantities that cannot be reliably quantified
"0" implies the complete absence of a compound

SC_ald5Δ									
**Time (h)**	**coumaran**	**Guaiacol**	**Benzenethanol**	**Benzeneacetic acid**	**Tyrosol**	**4-Vinylguaiacol**	**ortho-Vanillin**	**4-Hydroxybenzoic acid**	**p-Coumaric acid**
**0**	0	0	0	0	0	0	0.219 ± 0.01	0	9.587 ± 0.10
**12**	0	0	0	0	0	0	0.260 ± 0.03	0	8.454 ± 0.10
**24**	✓	0.678 ± 0.07	0.037 ± 0.004	0.131 ± 0.01	1.247 ± 0.15	0.102 ± 0.03	0.255 ± 0.07	0.084 ± 0.004	8.032 ± 0.10
**36**	0.218 ± 0.02	0.694 ± 0.02	0.910 ± 0.02	1.103 ± 0.50	1.120 ± 0.08	0.353 ± 0.03	0.337 ± 0.02	0.659 ± 0.07	7.067 ± 0.09
**48**	0.282 ± 0.02	0.203 ± 0.1	0.454 ± 0.04	0.316 ± 0.11	0.490 ± 0.08	0.216 ± 0.1	0.233 ± 0.02	0.089 ± 0.06	4.238 ± 0.05
**60**	0.107 ± 0.01	✓	1.067 ± 0.02	0	0.098 ± 0.01	0	0.205 ± 0.001	0	2.202 ± 0.02
**72**	0	0.073 ± 0.01	0.362 ± 0.12	0.197 ± 0.03	0	0	0.189 ± 0.04	0	0.701 ± 0.01
**96**	0	0	0.503 ± 0.08	0	0	0	0.332 ± 0.01	0	0

"✓" implies the presence of a compound in quantities that cannot be reliably quantified
"0" implies the complete absence of a compound

CTRL									
**Time (h)**	**coumaran**	**Guaiacol**	**Benzenethanol**	**Benzeneacetic acid**	**Tyrosol**	**4-Vinylguaiacol**	**ortho-Vanillin**	**4-Hydroxybenzoic acid**	**p-Coumaric acid**
**0**	0	0	0	0	0	0	0.293 ± 0.02	0	9.531 ± 0.20
**12**	0	0	0	0	0	0	0.263 ± 0.02	0	8.449 ± 0.16
**24**	✓	0.271 ± 0.03	0.022 ± 0.02	0.053 ± 0.001	1.311 ± 0.18	0.041 ± 0.01	0.281 ± 0.03	0.057 ± 0.005	8.026 ± 0.15
**36**	0.252 ± 0.02	0.788 ± 0.21	1.057 ± 0.28	1.245 ± 0.50	1.260 ± 0.1	0.395 ± 0.03	0.310 ± 0.05	0.754 ± 0.050	6.982 ± 0.13
**48**	0.238 ± 0.02	0.166 ± 0.08	0.371 ± 0.03	0.260 ± 0.09	0.398 ± 0.05	0.176 ± 0.02	0.278 ± 0.04	0.074 ± 0.005	4.153 ± 0.08
**60**	0.075 ± 0.07	✓	1.036 ± 0.42	0	0.147 ± 0.01	0	0.286 ± 0.02	0	2.157 ± 0.04
**72**	0	0.065 ± 0.01	0.267 ± 0.01	0.177 ± 0.03	0	0	0.269 ± 0.04	0	0.670 ± 0.01
**96**	0	0	0.567 ± 0.07	0	0	0	0.311 ± 0.03	0	0

"✓" implies the presence of a compound in quantities that cannot be reliably quantified
"0" implies the complete absence of a compound
